# Three, two, one! Revision of the long-bodied sphaerodorids (Sphaerodoridae, Annelida) and synonymization of *Ephesiella*, *Ephesiopsis* and *Sphaerodorum*

**DOI:** 10.7717/peerj.5783

**Published:** 2018-10-26

**Authors:** María Capa, Torkild Bakken, Karin Meißner, Arne Nygren

**Affiliations:** 1 Department of Biology, University of the Balearic Islands, Palma, Spain; 2 NTNU University Museum, Norwegian University of Science and Technology, Trondheim, Norway; 3 Forschungsinstitut Senckenberg, Deutsches Zentrum für Marine Biodiversitätsforschung, Hamburg, Germany; 4 Sjöfartsmuseet Akvariet, Göteborg, Sweden; 5 Institutionen för marina vetenskaper, Göteborgs Universitet, Göteborg, Sweden

**Keywords:** Diagnostic features, Polychaetes, Morphology, Molecular phylogeny, Reciprocal monophyly, Systematics, Classification, Genera synonimization

## Abstract

**Background:**

Long-bodied sphaerodorids (Annelida, Sphaerodoridae) is the common name for members of the three closely and morphologically homogenous currently accepted genera of benthic marine bristle worms: *Ephesiella*, *Ephesiopsis* and *Sphaerodorum*. Members of this group share the presence of two dorsal and longitudinal rows of macrotubercles with terminal papillae, and two longitudinal rows of microtubercles, features that are unique among sphaerodorids. Genera are distinguished by the chaetae morphology. Members of *Ephesiella* are characterised by having compound chaetae (except, sometimes, simple chaetae in the first chaetigers), *Sphaerodorum* bear only simple chaetae, and *Ephesiopsis* have both compound and simple chaetae in all parapodia.

**Methods:**

Mitochondrial (partial COI and 16S rDNA) and nuclear (partial 18S rDNA and 28S rDNA) sequence data of long-bodied sphaerodorids with compound and simple chaetae, and an outgroup of additional seven sphaerodorid species were analysed separately and in combination using Bayesian inference (BA), and Maximum Likelihood (ML) methods. Long-bodied sphaerodorids from around the world (including type specimens) were examined under a range of optical equipment in order to evaluate putative generic and specific diagnostic features, in addition to intraspecific variability.

**Results:**

Phylogenetic analyses of mitochondrial and nuclear DNA sequences of specimens identified as *Ephesiella* and *Sphaerodorum,* based on chaeta morphology, were performed. *Sphaerodorum* and *Ephesiella* were recovered as paraphyletic and nested within each other. Revision of current nominal species diagnostic features are performed and discussed.

**Discussion:**

Results contradict current generic definitions. Recovery of paraphyletic compound and simple chaetae clades urge the synonymization of these two genera of long-bodied sphaerodorids. Morphological data also suggest the synonymization of *Ephesiopsis*.

## Introduction

Sphaerodoridae is a group of marine annelids characterised by the presence of spherical tubercles over their surface. They are generally small in size (less than 2 mm, and less than 40 segments) and have an ellipsoid shape with a greatly convex dorsal surface. However, some species are characterised by having longer and slender bodies (2–50 mm, and 50–100 segments), parallel lateral sides, tapering on posterior segments and a slightly convex dorsal surface (Capa, Bakken & Purschke, 2014; Capa, Aguado & Bakken, 2016). Members of these long-bodied sphaerodorids have been ascribed to the genera *Ephesiella* Chamberlin, 1919, *Ephesiopsis* Hartman & Fauchald, 1971 and *Sphaerodorum* Ørsted, 1843.

Besides their resemblance in the overall appearance, the long-bodied sphaerodorids also share a number of additional morphological features, a reason why their taxonomic history has been convoluted (see Moore, 1909; Mileikovsky, 1967 and Fauchald, 1974 for summary on the matter). Unlike other sphaerodorids, the long-bodied species have dorsal microtubercles (small protuberances with a basal collar and a distal digitiform terminal papilla) forming two longitudinal rows along the dorsum with a single pair per segment. Microtubercles appear to be reduced dorsal cirri, homologous to those present in other errant polychaetes (Helm & Capa, 2015). Long-bodied sphaerodorids bear a pair of large macrotubercles per segment, forming longitudinal rows running in parallel and outside to the microtubercles, with an almost spherical shape and a distal digitiform papilla (e.g.,  Fauchald, 1974; Capa, Bakken & Purschke, 2014). Long-bodied sphaerodorids may also have modified stout and curved simple chaetae in the anterior chaetigers, also called hooks (e.g.,  Fauchald, 1974; Capa, Bakken & Purschke, 2014). Their presence seems to be species specific and has been reported in members attributed to all three genera (e.g.,  Hartman & Fauchald, 1971; Fauchald, 1974; Capa, Bakken & Purschke, 2014).

All other sphaerodorids, mainly short bodied, lack simple chaetae. Sphaerodorids have an axial muscular pharynx, similar to that in other Phyllodocida (e.g., Capa, Bakken & Purschke, 2014; Helm & Capa, 2015). In the short body forms a so called ‘proventricle’, similar in shape, but not in structure, to the analogous proventricle in Syllidae has been described in detail (Filippova et al., 2010). Recently, a less developed bulbous pharyngeal region was also described in the long-bodied sphaerodorids (Helm & Capa, 2015). The structures referred to as copulatory organs, found in some species of *Sphaerodoridium* Lützen, 1961, *Sphaerodoropsis* Hartman & Fauchald, 1971 and *Sphaerephesia* Fauchald, 1972 (Moreira, Cacabelos & Troncoso, 2004; Moreira & Parapar, 2012; Capa & Bakken, 2015; Capa & Rouse, 2015), have never been reported in members of the long-bodied genera.

The only attribute that has been used to classify long-bodied sphaerodorids into the three genera is the chaetal morphology. Members of *Ephesiella* are characterised by having compound chaetae (with a shaft and a blade), *Sphaerodorum* bear only simple chaetae (not divided), and *Ephesiopsis* both compound and simple chaetae in all parapodia (e.g., Hartman & Fauchald, 1971; Fauchald, 1974; Capa, Bakken & Purschke, 2014). However, in some *Ephesiella* species the compound chaetae seem to be pseudo-compound, with a distal end (equivalent to blades) distinguished from a proximal part (shafts) by an oblique indentation, but not completely split (e.g., Moore, 1909). Similarly, some species of *Sphaerodorum,* with typically simple chaetae, have a subtle groove resembling the pseudo-compound chaetae in *Ephesiella*. This has been interpreted as an indication of the fusion of blade and shaft (e.g.,  Martín & Alvà, 1988; Moreira & Parapar, 2011). The existence of pseudo-compound chaetae has not been given much attention (but see Fauvel, 1911). However, it is the main reason why the generic value of such a feature, and validity of the three genera are here being questioned.

**Figure 1:**
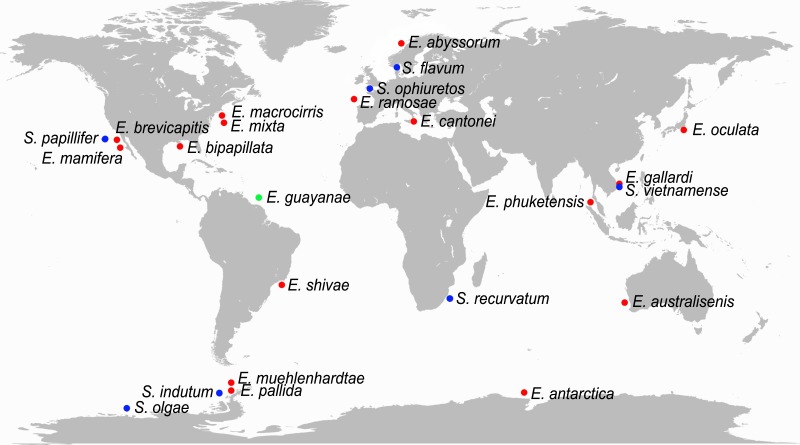
World map with type localities of accepted species of *Ephesiella* (red dots), *Ephesiopsis* (green dots) and *Sphaerodorum* (blue dots).

*Ephesiella* currently comprises 17 nominal species, *Sphaerodorum,* seven, and *Ephesiopsis,* one (Capa, Bakken & Purschke, 2014; Capa, Osborn & Bakken, 2016). *Ephesiella* and *Sphaerodorum* are cosmopolitan genera, with species reported from the Arctic and Antarctic oceans, tropical and temperate regions of the Atlantic and Indo-Pacific and from the intertidal to the deep sea (Fig. 1). Representatives of *Sphaerodorum* and *Ephesiella* are in most cases found in the same samples (Fauvel, 1911; Bakken et al., 2010 and M Capa, pers. obs., 2016), regardless of their geographic distribution or depth range. Species of each of these two groups have restricted geographic distribution, but some species have also been reported as widely distributed both in geographic and bathymetrical ranges. For example, *Sphaerodorum flavum* (Ørsted, 1843) has been recorded from the Arctic, North Atlantic (both American and European coats), Mediterranean Sea, South Africa and Japan between intertidal environments to 1,500 m depth (Webster & Benedict, 1887; Fauvel, 1923; Pettibone, 1963; Day, 1967; Imajima, 1969; Fauchald, 1974; Hartmann-Schröder, 1996; Kirkegaard, 2001). The dispersal capability of long bodied-sphaerodorids is largely unknown but the reproductive biology of *S. flavum* has been studied and fertilization has been interpreted to be external and larvae to have direct development (Christie, 1984). This information is in conflict with its reported distribution, since benthic organisms without planktonic larvae are supposed to have a limited dispersal capability (e.g., Barnes & Hughes, 1999). This is why some authors have indicated the need to revise of some of these occurrence records (Fauchald, 1974; Moreira, 2012). The presence of the same species in a broad geographical area and range of environments would not only involve at least great dispersal capabilities but also high physiological plasticity. Nevertheless, if the contrary is demonstrated, these groups could become another example of species complexes indicating that species discrimination is not always an easy task (Fauchald, 1974; Moreira, 2012; Nygren, 2014; Capa & Bakken, 2015).

Specific diagnostic features commonly used in the literature to distinguish between species of *Ephesiella*, *Ephesiopsis* and *Sphaerodorum* include: the presence and relative length of head appendages, the presence of eyes, the presence and number of hooks in anterior (generally first) chaetigers, the relative position of macro and microtubercles, the shape and relative length of the ventral cirri and the acicular lobe, the presence of pre- and postchaetal lobes, number and arrangement of parapodial papillae and chaetal morphology (e.g., Hartman & Fauchald, 1971; Fauchald, 1974; Hartmann-Schröder, 1982; Mòllica, 1994; Bakken, 2002; Rizzo, 2009; Moreira & Parapar, 2011; Capa & Bakken, 2015). The intraspecific variability has generally not been reported for species in the literature, and some of the cited characters have not been referred to in the descriptions. A revision of some of the species is therefore needed, but is outside of the scope of the present paper.

The aims of this study are: (1) to assess the monophyly of *Ephesiella*, *Ephesiopsis* and *Sphaerodorum;* (2) to test if the chaetal morphology supports the segregation of these groups and; (3) to test if the morphological features described in the literature are useful for species discrimination.

## Materials and Methods

### DNA sequence data

Mitochondrial (partial COI and 16S rDNA) and nuclear (partial 18S rDNA and 28S rDNA) sequence data of a set of 17 long-bodied sphaerodorid species (formerly considered as members of *Ephesiella* or *Sphaerodorum*) with compound or simple chaetae respectively, and an outgroup of additional seven species (members of other sphaerodorid genera: *Sphaerodoropsis*, *Clavodorum* and *Sphaerodoridium*) were analysed.

#### DNA extraction, PCR amplification and sequencing

DNA was extracted with QuickExtract DNA Extraction (Epicentre, Madison, WI, USA); a small piece, usually one or two parapodia, were put in 50–100 µl QuickExtract, and treated with 65 °C for 45 min followed by 2 min in 95 °C in a dry block thermostat. We used the primers 16SANNF (GCGGTATCCTGACCGTRCWAAGGTA) (Sjölin, Erseus & Källersjo, 2005) or 16SARL (CGCCTGTTTATCAAAAACAT), together with 16SBRH (CCGGTCTGAACTCAGATCACGT) (Palumbi, 1996) for 16S rDNA; LCO1490 (GGTCAACAAATCATAAAGATATTGG) and HCO2198 (TAAACTTCAGGGTGACCAAAAAATCA) (Folmer et al., 1994) for COI; 28SC1 (ACCCGCTGAATTTAAGCAT) and 28SD2 (TCCGTGTTTCAAGACGG) (Lê, Lecointre & Perasso, 1993) for 28S rDNA (D1–D2 region); and 18SAL (AACCTGGTTGATCCTGCCAGT and CCAACTACGAGCTTTTTAACTG), 18SBO (TGATCCTTCCGCAGGTTCACCT and AAGGGCACCACCAGGAGTGGAG), and 18SCY (CGGTAATTCCAGCTCCAATAG and CAGACAAATCGCTCCACCAAC) (Apakupakul, Siddall & Burreson, 1999), amplifying three overlapping fragments, for 18S rDNA. PCR mixtures contained 0.33 µl of each primer (10 µM), 1 µl of DNA template, and 10 µl of RedTaq 1.1 × MasterMix 2.0 mM MgCl2 (VWR). Temperature profile was as follows: 96 °C/1 min–(95 °C/30 s–52 °C (for COI, 16S rDNA, and 18S rDNA) or 60 °C (for 28S rDNA)/30s–72 °C/60 s) × 29 cycles–72 °C/7 min. PCR products were visualized with UV-light (312 nm) following electrophoresis for c. 15 min on a 1% agarose gel (1 g Agarose DNA Pure Grade (VWR) in a TAE Buffer Ultra Pure Grade (Amresco, Dallas, TX, USA)) containing 1 µl GelRed Nuclear Acid Stain (Bioticum, El Monte, CA, USA) in 50 ml agarose. Each PCR product was purified with 2 µl cleaning solution made from 500 µl mQ-H20, 40 µl FastAP (EF0651), 45µl Buffer FastAP, and 20 µl Exonuclease (EN0581) (Thermo Scientific, Waltham, MA, USA). PCR products with added cleaning solution were run for 37 °C in 60 min, followed by 75 °C in 15 min. Sequencing was performed at Eurofins Genomics, DNA Sequencing Department in Ebersberg, Germany.

Overlapping sequence fragments were merged into consensus sequences using Geneious version 7.0.6 available from Geneious 9 (http://www.geneious.com/). We used MAFFT v7.017 (Katoh et al., 2002) within Geneious 7.0.6 with the following settings: algorithm = E-INS-i, scoring matrix = 200PAM/*k* = 2, gap open penalty = 1.53 to align the sequences. We used the online GBlocks server v. 0.91b (Castresana, 2002), using the options ‘Allow gap positions within the final blocks’ and ‘Allow less strict flanking positions’, to detect alignment-ambiguous sites (Talavera & Castresana, 2007). Analyses were performed both with and without these alignment-ambiguous sites. Gene partitions were concatenated using Mesquite v. 2.75 (Maddison & Maddison, 2008). Information about the specimens used in DNA sequencing, vouchers and accession numbers can be found in Table 1.

**Table 1:** Collection information on the specimens used for the phylogenetic analyses, vouchers, and GenBank accession numbers. Registration code numbers refer to following institutions: SMF: Senckenberg Museum Frankfurt, ZMBN: Museum of Zoology at the University of Bergen, NTNU-VM: NTNU University Museum.

**TAXA**	**Indiv.**	**COI**	**16S**	**28SD1-D2**	**18S**	**Reg number**	**Locality**	**Depth (m)**
*Clavodorum atlanticum*	SPH019	KR019912	KR019947		KR019897	SMF 23901	Argentinian Basin****	4,607
*Sphaerodoropsis martinae*	SPH021	KR019880	KR019944	MH768889	KR019909	SMF 21466	Argentinian Basin	4,607****
*Sphaerodoropsis* cf. *longianalpapilla*	SPH023	KR019888	KR019944	MH768892	KR019906	SMF 23912	Argentinian Basin	4,607
*Sphaerodoropsis philippi*	SPH089	KR019893	KR019938	MH768890	KR019902	ZMBN 103141	Norwegian Sea****	1,315
*Sphaerodoropsis* sp. 4	SPH297	MH768926	MH768961	MH768891	MH768912	ZMBN 125432	Skagerrak	238
*Sphaerodoridium fauchaldi*	SPH312	MH768944	MH768963	MH768888	MH768911	ZMBN 125433	Kvitsøy, Norway	58
*Sphaerodoridium minutum*	SPH316	MH768943	MH768962	MH768893	MH768923	ZMBN 125434	Skagerrak	275
*Sphaerodorum* sp.	SPH138	MH768933	MH768957	MH768901	MH768915	ZMBN 125431	Barents Sea, Norway	217
*Sphaerodorum* sp.	SPH247	MH768934	MH768958	MH768902	MH768916	ZMBN 115538	Svalbard	74
*Sphaerodorum* sp.	SPH072	MH768932	MH768956	MH768900	MH768914	ZMBN 125430	Sogn, Norwegian Sea	103
*Ephesiella sp.*	SPH078	MH768935	MH768959	MH768903	MH768917	SMF 24694	Iceland Basin, S Iceland	2,749
*Ephesiella sp.*	SPH162	MH768936	MH768960	MH768904	MH768917	SMF 24695	Iceland Basin, S Iceland	2,749
*Sphaerodorum* sp.	SPH012	MH768930	MH768954	MH768898	KR019915	ZMBN 125429	Norwegian Sea	219
*Sphaerodorum* sp.	SPH224	MH768931	MH768955	MH768899	MH768913	ZMBN 115515	Trøndelag, Norwegian Sea	319
*Ephesiella* sp.	SPH203	MH768937	MH768946	MH768905		ZMBN 115494	Banyuls, W Mediterranean	25
*Ephesiella* sp.	SPH204	MH768938	MH768947	MH768906		ZMBN 115495	Banyuls, W Mediterranean	25
*Ephesiella* sp.	SPH045	MH768941	MH768948	MH768907	MH768924	SMF 24631	Irminger Basin, S Iceland	1,594
*Ephesiella* sp.	SPH080	MH768942	MH768949	MH768908	MH768925	SMF 24632	Irminger Basin, S Iceland	1,594
*Ephesiella* sp.	SPH207	MH768939	MH768952	MH768909	MH768921	ZMBN 115498	Skagerrak	406
*Ephesiella* sp.	SPH212	MH768940	MH768953	MH768910	MH768922	ZMBN 115503	Barents Sea, Norway	304
*Ephesiella* sp.	SPH011	KR019884	KR019942	MH768894	KR019929	ZMBN 125428	Norwegian Sea	823
*Ephesiella* sp.	SPH155	MH768927	MH768945	MH768895	MH768919	DZMB HH 30510	Denmark Strait, E Greenland	1,248
*Ephesiella* sp.	SPH164	MH768928	MH768950	MH768896		NTNU-VM 73254	Agdenes, Norway	45–107
*Ephesiella* sp.	SPH232	MH768929	MH768951	MH768897	MH768920	ZMBN 115523	Skagerrak	30

#### Phylogenetic analyses

The mitochondrial (COI and 16S rDNA and nuclear data sets (18S rDNA and 28S rDNA) were analysed separately and combined using Bayesian inference (BA), and Maximum Likelihood (ML). Bayesian analyses (BAs) of separate and combined data sets were run in MrBayes 3.2 (Ronquist & Huelsenbeck, 2003), and the best-fit models were selected using the Akaike information criterion in JModel (Darriba et al., 2012). The protein coding gene COI was further divided into two partitions, one with the first and second positions, and one with the third positions. The selected best-fit models were a general time reversible model with gamma distributed rate across sites and a proportion of the sites invariable (GTR+I+G) for the COI-partition with first and second positions, 16S rDNA, 18S rDNA, and 28S rDNA, while a Hasegawa, Kishino and Yano model, with gamma distributed rate across sites (HKY+G) was selected for the COI-partition with third positions.

Partitions were unlinked for the parameters statefreq, revmat, shape and pinvar. Rateprior for the partition rate multiplier was set to be variable. Number of generations was set to three million, with four parallel chains (three hot, one cold), sample frequency was set to 1,000, and number of runs set to two. One fourth of the samples were discarded as burn-ins. Maximum likelihood analyses (MLs) were performed in raxmlGUI (Silvestro & Michalak, 2012). In RAxML, the analyses were run with the GTRGAMMAI model, the combined data set was partitioned as in BA, and clade support was assessed using 1000 bootstrap replicates.

### Source of material

A comprehensive number of specimens from several localities around the world were examined in order to evaluate the variation of the established and other potential generic features, in addition to the specific diagnostic features and intraspecific variability. Since species boundaries are not clear, intraspecific variability is the morphological variation found among paratypes or specimens collected near the type locality. Comparisons between the literature and personal observation were also made (Supplemental Information) and are herein discussed.

Access to the following museum collections have allowed the revision of the type material of all available species and examination of additional non-type material: NTNU University Museum, Norwegian University of Science and Technology, Trondheim (NTNU-VM); Natural History Collections, University of Bergen Bergen (ZMBN); Akvaplan-Niva, Tromsø (AN); Museo Nacional de Ciencias Naturales, Madrid (MNCN); Museum National d’Histoire Naturelle, Paris (MNHN); ZMH Hamburg; Deutsches Zentrum für Marine Biodiversittätsforschung, Hamburg (SMF, DZMB); Australian Museum, Sydney (AM); Museum of Evolution, Uppsala University, Uppsala (UPSZ); Museum of Natural History, London (NHM); Natural History Museum of Los Angeles County, Los Angeles (LACM); Museu de Zoologia da Universidade Estadual de Campinas, Campinas (ZUEC); National Museum of Natural History, Smithsonian Institution, Washington (USNM); Phuket Marine Biological Center, Phuket (PMBC); Museum Victoria, Melbourne (MV); Museum and Art Gallery of the Northern Territory, Darwin (MAGNT).

Specimens were examined under a range of optical equipment in different institutions. Photographs were taken with a Leica DFC420 camera attached to a Leica MZ16A light microscope. Stacks of multi-focus shots were merged into a single photograph to improve resolution with Leica Application Suite v3.7 software (Leica Microsystems, Wetzlar, Germany). Other photographs were taken with a Dino-Eye camera attached to the microscopes and run with the Dino Capture 2.0 software (Dino-Lite Digital Microscope). Parapodia were mounted on slides with glycerol for observation of papillae and chaetae from different angles. Some specimens were dehydrated in ascending concentration of ethanol and then critical-point dried. The prepared samples were mounted on holders and sputter-coated with gold (10 nm thickness). The micromorphology and topography were determined using a Philips FEI INSPECT (Hillsboro, OR, USA) Scanning Electron Microscope (SEM) at MNCN, Madrid. The samples were observed with the Back Scattering Electron Detector (BSED) with a resolution at high vacuum of 4.0 nm at 30 kV.

## Results

### Assessing the monophyly of *Ephesiella*, *Ephesiopsis* and *Sphaerodorum*

The combined data set of COI, 16S rDNA, 18S rDNA, and 28S rDNA consists of 4,137 characters, divided on the genetic markers as 658, 480, 2,069, and 930 characters; excluding alignment-ambiguous sites data set consists of 3,526 characters divided on the genetic markers as 658, 435, 1,689, and 744 characters.

**Figure 2:**
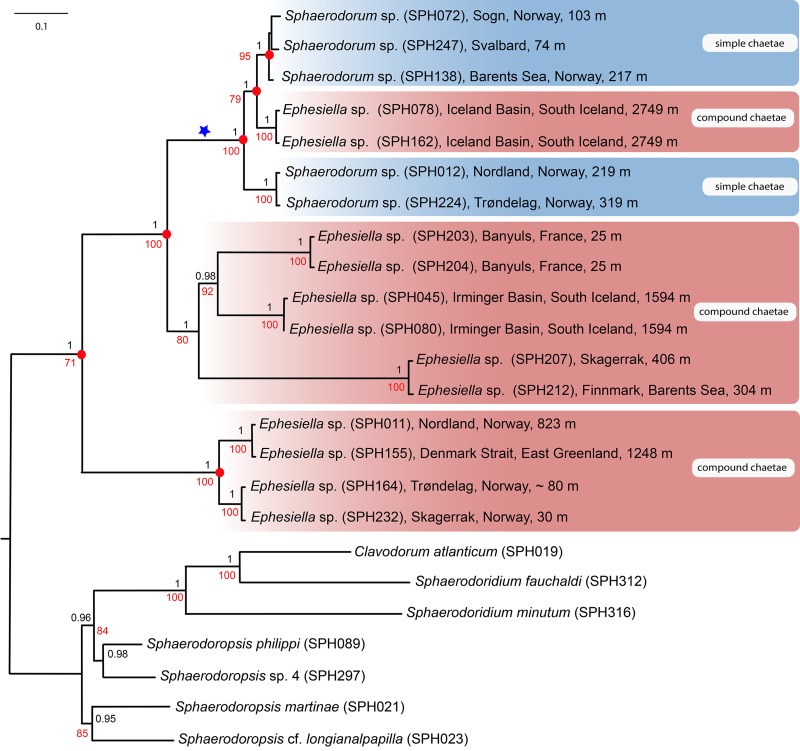
Maximum likelihood topology of the nuclear and mitochondrial DNA sequence data combined. Bootstrap supports shown on nodes in red, boostrap values of congruent noted after Bayesian analyses in black. Scale bar, average of nucleotide substitutions per site.

Majority-rule topologies from BA and ML of the separate (i.e., mitochondrial and nuclear) as well as for the combined data set are congruent, but slightly more resolved in the BA analyses. Excluding alignment-ambiguous sites only affected outgroup relationships, did not change ingroup relationships, and only had minor effect on support values (±0.05 posterior probability, ±5 in bootstrap support), except for the monophyly of ingroup which increased from 71 in the ML-analysis for the combined mitochondrial and nuclear data set, to 100 in the ML-analyses excluding alignment-ambiguous sites. Tree topology from the combined data set including all sites is shown in Fig. 2. Resulting trees from all other analyses can be found in the Supplemental Information. Analyses of the combined dataset provided identical results to the analyses of nuclear data; results from the mitochondrial data set differ in the position of specimens SPH203, SPH204 (alternative placement shown with blue star in Fig. 2), but these have low support (0.56, and 0.74 posterior probabilities in BA, <50 bootstrap support in ML) for alternative nodes. Ingroup nodes that are found in both separate data sets as well as the combined data set is marked with filled red circle (Fig. 2).

Results unequivocally show that neither *Sphaerodorum* (blue clades, Fig. 2) nor *Ephesiella* (red clades, Fig. 2) are monophyletic. A clade with specimens bearing compound chaetae (namely *Ephesiella*) was found nested within a clade of simple chaetae (*Sphaerodorum*) that is, simultaneously, nested within specimens assignable to *Ephesiella* (Fig. 2). Revision of type material and additional specimens of several species revealed that intermediate forms, pseudo-compound chaetae, are found among members of the three genera and puts into question the legitimacy of this feature to discriminate between the groups and also the validity of the genera themselves.

Specimens (initially identified as members of *Ephesiella abyssorum* (Hansen, 1879) and *Sphaerodorum flavum* (Ørsted, 1843) from North East Atlantic and Norwegian Sea were recovered in different clades, separated by long genetic distances (Fig. 2), indicating the potential existence of several species in this area.

**Figure 3:**
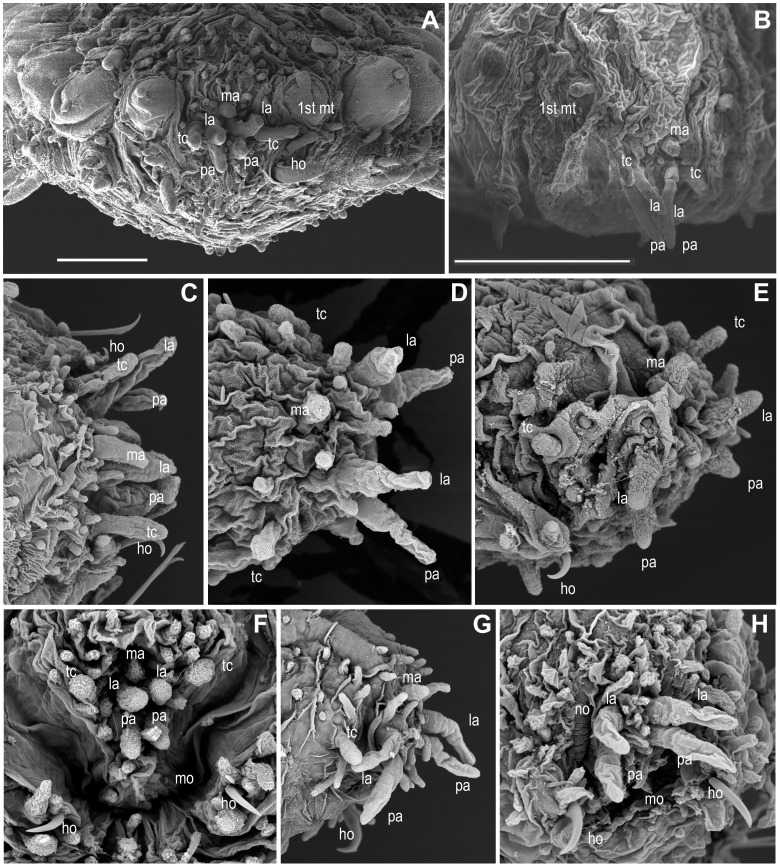
Anterior end and prostomial appendages. (A) *Ephesiella* cf. *abyssorum* from Nordland, Norway (ZMBN 126045); (B) *Ephesiella* cf. *abyssorum* from Brittany, France (MNHN A421); (C) *Ephesiella abyssorum* from off Nordland, Norway (NTNU-VM 73707); (D) *Ephesiella* cf. *cantonei* from Chafarinas Islands (MNCN 16.01/3450); (E) *Ephesiella antarctica*, from Livingston Island, Antarctica (MNCN 16.01 3555); (F) *Sphaerodorum flavum* from Kvamsøya, Norway (ZMBN 125840); (G) *Sphaerodorum flavum*, A Coruña, Spain (MNCN 16.01/13265); (H) *Sphaerodorum flavum*, A Coruña, Spain (MNCN 16.01/13265).

### Revision of traditional species diagnostic features

The traditional attributes used for species discrimination in the literature, and the observations after revision of type and non-type specimens are described below, and comparative tables are provided in order to check the intraspecific variability and the validity of these features (Supplemental Information). The terminology has also been reviewed. In this paper we are proposing the synonimization of *Ephesiella*, *Ephesiopsis* and *Sphaerodorum*, but the names used in the following section are kept as hitherto accepted to enable a good understanding of the comparison of features between members of the different species and genera.

### Head appendages (Figs. 3A–3H, Supplemental Information).

Members of *Ephesiella*, *Ephesiopsis* and *Sphaerodorum,* like most other sphaerodorids, share the presence of seven head appendages, herein referred to as palps (ventral-most pair), lateral antennae (dorsal-most pair in prostomium) and median antenna (single dorsal-most appendage), and a pair of tentacular cirri (Figs. 3A– 3H), as this is consistent with generic diagnoses (Hartman & Fauchald, 1971; Fauchald, 1974; Capa, Bakken & Purschke, 2014). Some species were, nevertheless, described as lacking the lateral antennae (*Ephesiella pallida* Fauchald, 1974) or without the median antenna (*E. cantonei* Mòllica, 1994; *E. ramosae* Desbruyères, 1980) and *Sphaerodorum vietnamense* (Fauchald, 1974), conditions that have been considered as diagnostic for all of them, and justified their description as distinct species.

Revision of the type specimens of these four species did not allow verifying these attributes due to the condition of the material. In these specimens, the prostomium, or part of it, was retracted and the prostomial appendages are consequently hidden (e.g., Fig. 3F), or the prostomial papillae resemble in shape and size these sensory appendages, making the distinction between them difficult (e.g., Figs. 3D, 3F, 3H). Therefore, the real absence of these appendages in specimens is questioned. These four species were described based on single specimens and these attributes have not been reported since in specimens collected nearby type localities. Prostomial appendages are often all similar in size and shape, but the median antenna is slightly shorter or even spherical in some species (Figs. 3A, 3D, 3F). Relative length of appendages is reported to be a specific diagnostic feature but contraction due to fixation and preservation has not been studied nor considered in the literature, and variation within some populations has been observed in this study (Figs. 3G, 3H; Supplemental Information).

**Figure 4:**
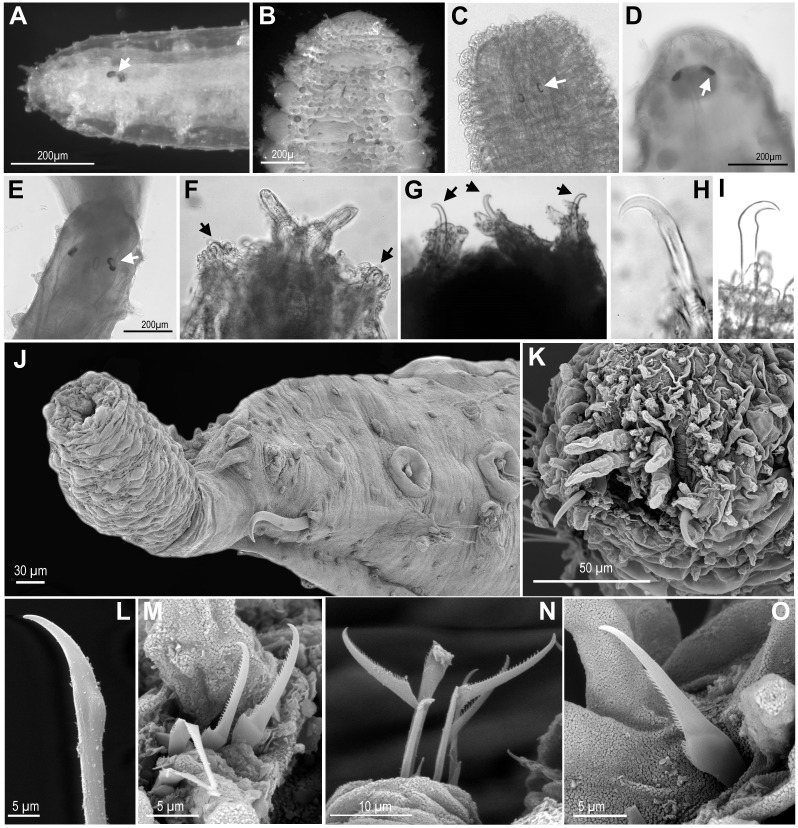
Eyes, and first chaetiger hooks (recurved simple chaetae) and chaetae. (A) *Ephesiella* cf. *cantonei*, from Banyuls, France (LACM AHF POLY 5372); (B) *Sphaerodorum flavum* from Greenland (ZMBN 126043); (C) *Ephesiella phuketensis*, holotype (PMBC 18542); (D, E) *Sphaerodorum* sp. Canada, Artic Ocean (USNM 35939); (F). *Ephesiella mixta*, holotype, North West of Bermuda (LACM AHF POLY 940); (G) *Ephesiella antarctica*, from Weddell Sea, Antarctica (USNM 46565); (H), *Ephesiella macrocirris,* holotype, New England, USA (LACM AHF POLY 936); (I), *Sphaerodorum papillifer* from California, USA (LACM AHF POLY 1284); (J). *Ephesiella abyssorum* from Kvamsøya, Norway (ZMBN 125840); (K), *Sphaerodorum flavum* from A Coruña, Spain (MNCN 16.01 13265); (L), *Ephesiella abyssorum* from Nordland, Norway (NTNU-VM 73707); (M). *Ephesiella* cf. *cantonei* from Málaga, Spain (MNCN 16.01/3450); (N), *Ephesiella* cf*. cantonei* from Málaga, Spain (MNCN 16.01 3448); (O), *Sphaerodorum flavum* from A Coruña, Spain (MNCN 16.01/13265).

The presence of an additional pair of appendages, often referred to as antenniform papillae, has been reported in some individuals of *Sphaerodorum olgae* (Moreira & Parapar, 2011). The taxonomic usefulness of this attribute is uncertain since the nature of these appendages and its function is still under discussion (e.g., Capa, Bakken & Purschke, 2014) and antenniform papillae do often resemble other head papillae. Its absence and presence show intraspecific variability and could also be considered present in individuals of several species.

#### Eyes (Figs. 4A–4E, Supplemental Information).

The cerebral eyes in sphaerodorids are attached to the brain and observed beneath the epithelium when this is translucent (Figs. 4A, 4C– 4E, Capa, Bakken & Purschke, 2014). They are often pushed back from the prostomium to as far as the second or third chaetiger (Fig. 4C; Capa, Bakken & Purschke, 2014). They are generally crescent-shaped (semicolon-shaped) spots (Figs. 4C–4E) and have been reported as absent or present in numbers of two, four or more, and colour varying from brown to red (in live specimens) and this variation has been considered as species specific (e.g., Fauchald, 1974; Mòllica, 1994; Hartmann-Schröder & Rosenfeldt, 1988; Imajima, 2003). Eyes have been observed even in individuals collected in hundreds of meters deep (e.g., in *Ephesiella abyssorum* (Hansen, 1879) from Norway, M Capa, pers. obs., 2016). In fixed, opaque specimens, eyes are often not conspicuous or seem to be absent (e.g., Fig. 4B) and variation to this condition has been observed among individuals of the same population and after longer preservation periods (e.g., when revisiting type material, Supplemental Information). The taxonomic value of presence and absence of eyes, colour, and arrangement should be assessed in live material when the epithelium is translucent. It is obvious that they are not always visible in fixed specimens and that the eye pigment could fade after some time as of all the types with reported eyes revisited had no obvious eyes, except for *E. muhlenhardtae* Hartmann-Schröder & Rosenfeldt, 1988. Species that were described as lacking eyes include *Ephesiella australiensis* and *Ephesiopsis shivae* (Rizzo, 2009)*,* yet in other descriptions the absence or presence of eyes was not mentioned Supplemental Information.

#### Hooks in anterior chaetigers (Figs. 4F–4K, Supplemental Information).

The function of hooks, anterior stout and curved simple chaetae, is unknown. However, its absence or presence has been one of the main diagnostic features for species discrimination (e.g., Fauchald, 1974; Mòllica, 1994; Hartmann-Schröder, 1982; Imajima, 2003). These structures are often drawn-out of the parapodia and are visible from the lateral or ventral side of individuals (Figs. 4F–4K) but they are sometimes withdrawn, and difficult to observe in fixed and opaque specimens (e.g., Bakken, 2002). Nevertheless, some translucent individuals, or specimens where parapodia have been dissected, seem to lack these chaetae, and this is not related to the gender or size of individuals (M Capa, pers. obs., 2016). The only known specimen of *Ephesiella mixta* (Hartman & Fauchald, 1971), with nine chaetigers, and the holotype of *Sphaerodorum ophiurophoretos* Martín & Alvà, 1988, with eight chaetigers bear hooks in the first chaetiger (Fig. 4F). Similarly, adult females and males of what has traditionally been considered *E. abyssorum* and *S. flavum* have been observed with and without this type of simple chaetae, so a sexual dimorphism is discarded. Further investigations should verify if the presence and arrangement of hooks in anterior chaetigers are attributes able to discriminate between lineages of this supposed complex of species (*E. abyssorum* + *S. flavum*; as shown in Fig. 2).

Hooks can also vary in number. Some specimens bear the typical pair, present in the first chaetiger, while others have another one or two sets of hooks in same parapodia, and the number can vary between the left and right parapodia (e.g., Bakken, 2002). The position of these hooks are generally attributed to the first chaetiger where records of other chaetae have not been reported in their absence or in addition to them. This is the most common arrangement (Figs. 3C, 3E, 3F, 4F–4K). However, a couple of individuals are herein reported bearing hooks in the first two chaetigers (Fig. 4G). The taxonomic importance of this variation is unknown, but may not be species specific.

**Figure 5:**
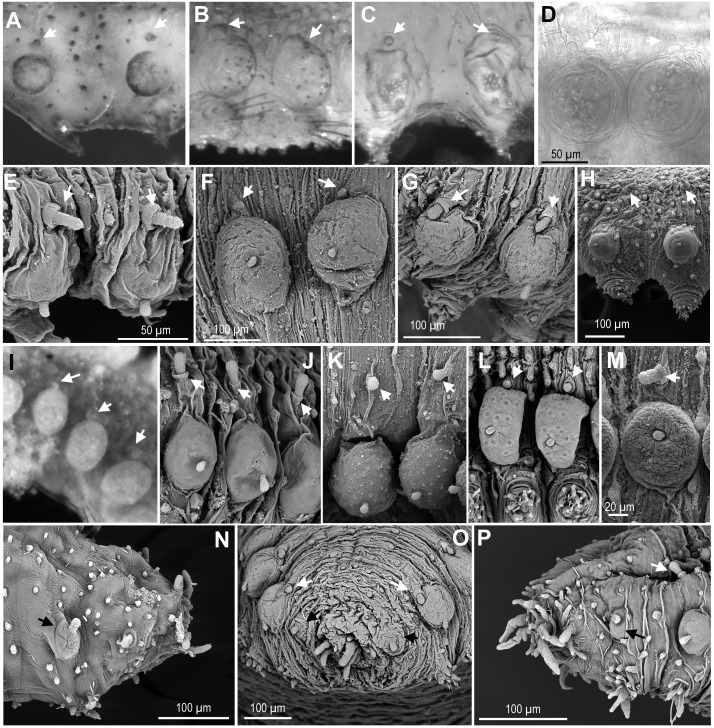
Macro and microtubercles. (A) *Ephesiella brevicapitis,* from California, USA (LACM AHF POLY 5389); (B) *Ephesiella* cf. *mammifera* from Baja California, Mexico (LACM AHF POLY 5413); (C) *Ephesiella* cf. *oculata*, from Japan (UUZM 54541); (D) *Ephesiella* sp. from Papua New Guinea (USNM 142064); (E) *Ephesiella australiensis* from Arafura Sea, Australia (AM W20663); (F) *Ephesiella* sp. from Brazil (ZUEC POL 4278), originally identified as *Ephesiopsis shivae*; (G). *Ephesiella* sp. from Brazil (ZUEC POL 4270); (H) *Ephesiella abyssorum* from Norway, (ZMBN 125842); (I) *Sphaerodorum indutum*, holotype, from South Shetland Islands, Antarctica (USNM 58481); (J) *Sphaerodorum flavum* from North West territories, Canada, (USNM 35939); (K) *Sphaerodorum flavum* from A Coruña, Spain (MNCN 16.01.13265); (L) *Sphaerodorum flavum* from the Oslofjord, Norway (ZMBN 125840); (M) *Sphaerodorum flavum* Norway (ZMBN 126046); (N), *Ephesiella* sp. from Antarctica (ZMH ANTXV13D48); (O), *Ephesiella* sp. from Rio de Janeiro, Brazil (ZUEC POL 4278); (P), *Sphaerodorum flavum* from A Coruña, Spain (MNCN 16.01/13265).

Some specimens of different putative species bear additional chaetae in the first chaetiger in addition to the hooks, described herein for the first time. These chaetae are not necessarily compound even in the typical “*Ephesiella*” species but mainly look pseudo-compound as they have only a groove indicating a possible fusion of shaft and blade (Figs. 4L–4O).

#### Relative position of macro- and microtubercles (Fig. 5, Supplemental Information).

The presence of dorsal macro- and microtubercles is characteristic of all members of the three genera of long-bodied sphaerodorids but variation within its relative position has been considered of taxonomic importance at species level. *Ephesiella mammifera* is the only species described with microtubercles partially fused to the dorsal edge of the macrotubercles (Fauchald, 1974). This condition was only verified in some segments of the paratypes (Fig. 5B), but not in the holotype which seemed to present macro- and microtubercles separated by a small gap. Moreover, some individuals belonging to other species also presented very close macro and microtubercles, or in contact (Figs. 5B–5G, 5I, 5L). This is the case for the holotype of *S. indutum* (Fig. 5I), and specimens of *Ephesiella* cf. *oculata* from Japan (Fig. 5C), *Ephesiella* spp. from Papua New Guinea and Brazil (Figs. 5D and 5F, respectively), *E. phuketensis* (*sensu* Bakken, 2002) and *S. flavum* from Norway (Fig. 5L), among others (see more examples in Figs. 5A–5L). Since specimens with this condition (macro- and microtubercles in close proximity) showed also some variation along the body, it is probable that the gap between macro and microtubercles can vary with the contraction of the tegument or the turgor of the macrotubercle (probably due to the amount of glandular content or preservation method).

**Figure 6:**
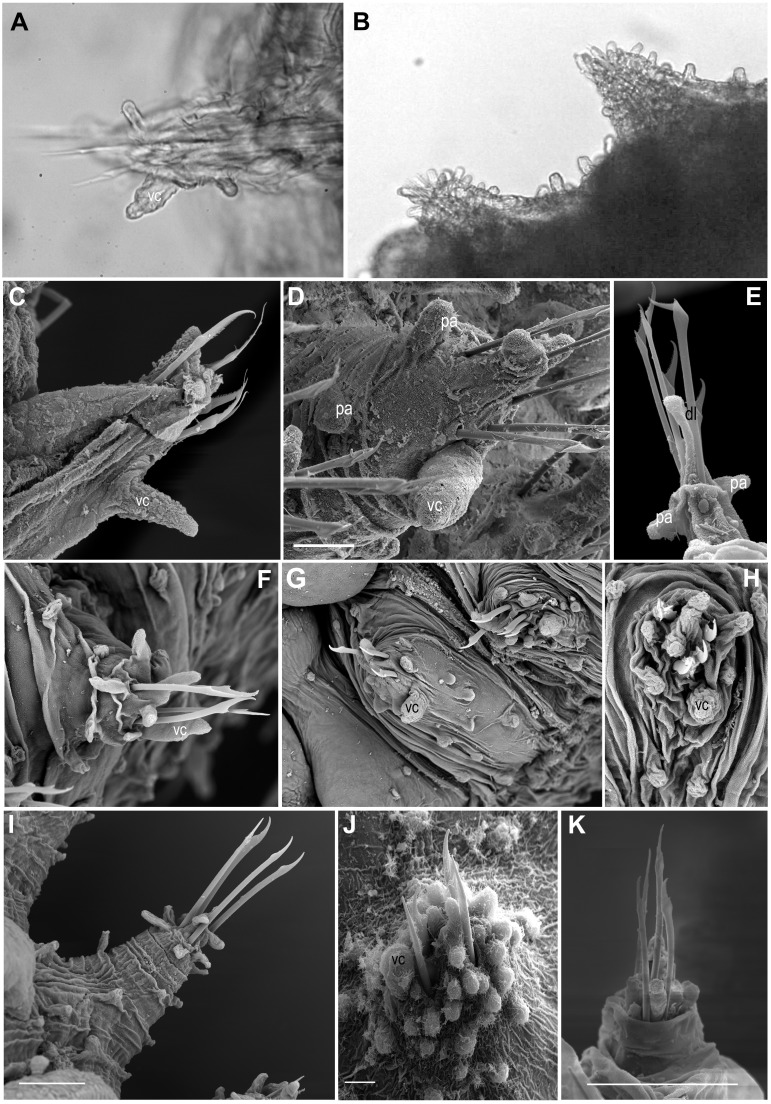
Parapodia and appendages. (A) *Ephesiella mixta*, holotype, North West of Bermuda (LACM AHF POLY 940); (B) *Ephesiella mulenhardtae*, paratype (ZHM P19150); (C) *Ephesiella* cf. *antarctica* from South Shetland Islands, Antarctica (MNCN 16.01/3555); (D) *Ephesiella abyssorum* from Nordland, Norway (ZMBN 126045); (E) *Ephesiella* cf. *cantonei* from Chafarinas Islands, Mediterranean Sea (MNCN 16.01.3455); (F) *Sphaerodorum flavum* from A Coruña, Spain (MNCN 16.01/13265); (G) *Sphaerodorum flavum* from Greenland (ZMBN 126044); (H) *Sphaerodorum flavum* (ZMBN 125840), (I) *Sphaerodorum flavum* from Norway (NTNU-VM 73708); (J) *Sphaerodorum flavum* from Brittany, France (MNHN A421); (K) *Ephesiella* cf. *brevicapitis* from California, USA (LACM AHF POLY 5390).

In most of the specimens examined, the first chaetiger lacks microtubercles and has an undersized macrotubercle (Figs. 5N–5P). This is not the case for the holotype of *E. mixta* and *E. muhlenhardtae* and the paratype of *E. australiensis* (Capa & Bakken, 2015) that have both types of tubercles in the first segment (bearing the hooks). The paratypes of the latter were in poor condition and almost disintegrated when examined and this feature, that could be of taxonomic value, was not corroborated, but should be considered and tested in future taxonomic revisions. Differences in the absence/presence of microtubercles on the first chaetiger have been observed even within the type material of *E. australiensis* (the holotype lacks microtubercles on first chaetiger), so it could well be a character showing intraspecific phenetic variation.

#### Shape and size of parapodia (Figs. 6A–6K)

The size and shape of parapodia can fluctuate considerably with contraction of parapodial muscles. Parapodial retractor muscles together with chaetal flexor muscles and acicular retractor muscles fill the whole parapodia (Helm & Capa, 2015) and are responsible for their size and shape. Differences of four times in length have been measured within the same fixed specimen, observation that has been verified in live specimens. Consequently, it does not seem appropriate to use the shape and length of these appendages for species diagnoses and probably not referring to its length when comparing relative length of other structures (i.e., ventral cirri, lobes, papillae, etc.).

One of the differences justifying the description of *Sphaerodorum papillifer* Moore, 1909 was its large parapodia with parallel sides (Moore, 1909; Fauchald, 1974), a feature with questionable taxonomic utility.

#### Ventral cirri (Figs. 6A–6K)

The shape, position and the length of the ventral cirri in relation to the acicular lobe have been considered diagnostic features for some long-bodied sphaerodorid species (e.g., Fauchald, 1974). Most descriptions refer to the ventral cirri as digitiform (Fauchald, 1974; Rizzo, 2009), basally inflated (Kudenov, 1987), with a distal protuberance (Moreira & Parapar, 2011) or with a distal articulation (as in *Ephesiopsis guayanae* (Hartman & Fauchald, 1971)) but direct re-examination of specimens from different species revealed that in most cases the ventral cirri are simple (without a distal articulation), with a wider base and with a distally thinner end resembling a bottle or a bowling pin (Figs. 6A, 6C, 6F–6H). Some exceptions have been found in small specimens or in anterior body segments of longer specimens, where the cirri are simply digitiform (cylindrical with a rounded tip; e.g., Figs. 6D–6J). In the single known specimen of *E. gallardoi* (Fauchald, 1974), cirri are distinctly conical (not mentioned in the original description), a feature which taxonomic value should be corroborated when intraspecific variability can be checked.

Ventral cirri slightly projecting beyond the parapodia is the general rule (*E. antarctica, E. brevicapitis* and *E. mixta,* e.g., Figs. 6D, 6F, 6G) except in anterior segments when it is usually shorter (e.g., Fig. 6C). However, a few species have been described as having shorter ventral cirri (e.g., Moore, 1909; Hartman & Fauchald, 1971; Fauchald, 1972; Fauchald, 1974). This feature may not be relevant for species discrimination since some intraspecific variation has also be noted and could be attributed to the degree of contraction of parapodia. The insertion of the cirri on the parapodia could be a valid character for distinguishing species, but again, its display could be affected by the level of contraction of parapodia. Revision of the types of *E. mixta* and *E. gallardoi* confirmed that the ventral cirri are inserted in the middle of the parapodia while in the rest of the species of *Ephesiella*, *Ephesiopsis* and *Sphaerodorum* ventral cirri have a more distal position (Figs. 6A, 6C, 6D, 6F–6H, 6J; Hartman & Fauchald, 1971; Fauchald, 1974; Rizzo, 2009; Moreira & Parapar, 2011).

#### Parapodial lobes (Figs. 6A–6K)

The sphaerodorid descriptions often refer to parapodial lobes, conceived as parapodial appendages that are longer than papillae, absent or present. The most common terms found in the literature are acicular lobe (not often described but assumed to be present in all specimens as it is housing the tip of the acicula), pre- and postchaetal lobes (implicitly located anterior and posterior to the chaetae, respectively) and dorsal chaetal lobe (implicitly located dorsally) (e.g., Fauchald, 1974). Several issues emerge from this classification of parapodial lobes. Chaetae are, in members of the long-bodied sphaerodorids, not arranged in a well-defined transverse row but in randomly arranged groups and interspersed with the almost indistinguishable lobes and distal papillae (Figs. 6C–6K), so the terms pre- and postchaetal lobes are here considered imprecise. This could explain why the description of specimens from same species are not always congruent in this matter. The tip of the acicula is sometimes difficult to spot and some authors may have referred to it as a terminal papillae or dorsal chaetal lobe (e.g., Fauchald, 1974; Moreira & Parapar, 2011). Some specimens clearly present a longer papilla on their dorsal distal end (e.g., Fig. 6E), but this is not always recognisable in all segments.

According to Fauchald (1974), sphaerodorid parapodial lobes differ histologically from the parapodial papillae, but at least in the long-bodied forms the papillae and lobes are difficult to distinguish externally (e.g., Figs. 6A–6K) and with the exception of the tip of the parapodia (i.e., the acicular lobe), that show strong neuronal innervation, indicating a sensory function, the nature and internal structure of other parapodial structures seem to be similar in all cases. All epithelial papillae (excluding macro- and microtubercles) seem to have a sensory function as they are provided with distinct ciliation, pores and lack glandular content (Helm & Capa, 2015). Since the lobes are difficult to tell apart from papillae in long bodied sphaerodorids, and do not seem to be recognised even after histological sections, its use is discouraged.

**Figure 7:**
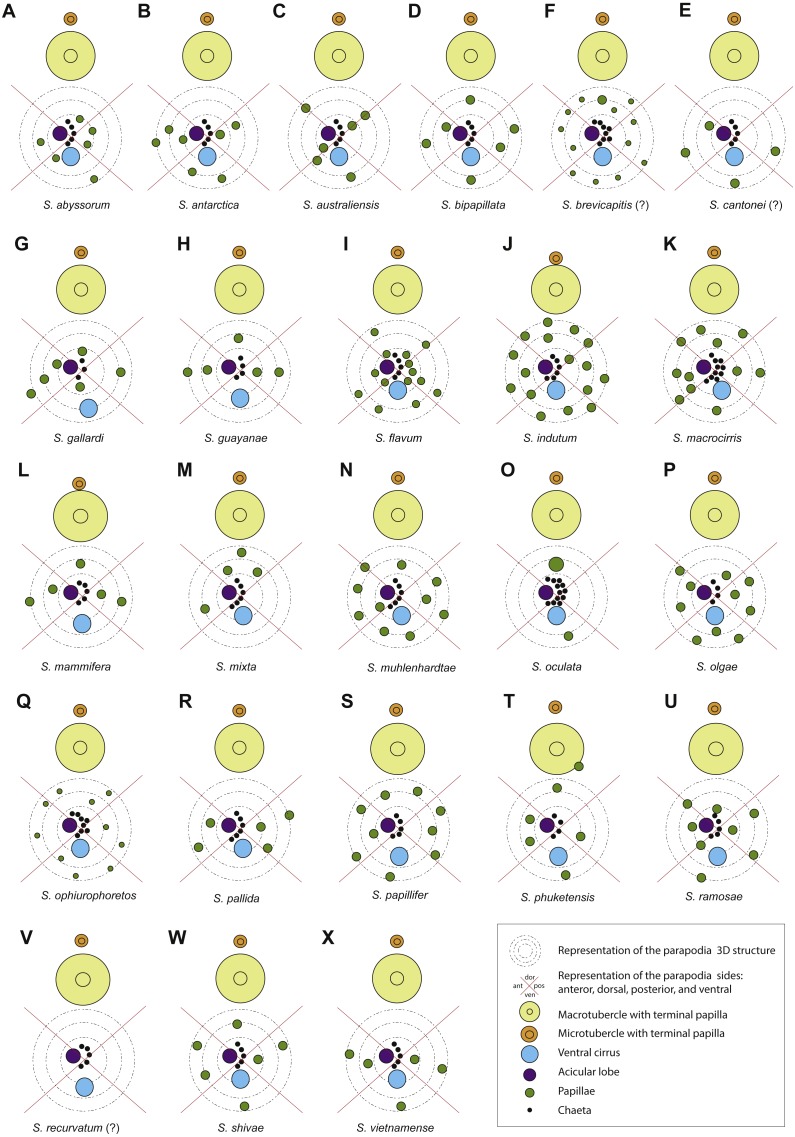
Schematic representation of parapodia of midbody segments, appendices, parapodial papillae and adjacent macro and microtubercles of type material, or additional material (with question marks). Symbols and colour codes shown in figure. (A) *Sphaerodorum abyssorum*, (B) *S. antactica*, (C) *S. australiensis*, (D) *S. bipapillata*, (E) *S. brevicapitis*, (F) *S. cantonei* (?), (G) *S. gallardi*, (H) *S. guayanae*, (I) *S. flavum*, (J) *S. indutum*, (K) *S. macrocirris*, (L) *S. mammifera*, (M) *S. mixta*, (N) *S. muhlenhardtae*, (O) *S. oculata*, (P) *S. olgae*, (Q) *S. ophiuretos*, (R) *S. pallida*, (S) *S. papillifer*, (T) *S. phuketensis*, (U) *S. ramosae*, (V) *S. recurvatum* (?), (W) *S. shivae*, (X) *S. vietnamense*.

#### Parapodial papillae (Figs. 6A–6K, 7)

The number and arrangement of parapodial papillae are features often used as diagnostic in sphaerodorid species identification. Nevertheless, a few descriptions indicate the intraspecific variability and it is not known if number or shape change with age and size or if papillae are retractile (as mentioned by Moore, 1909). The terminology used to refer to the arrangement of these papillae in the parapodia is ambiguous. On one hand, descriptions refer to the number of papillae per side of parapodium (a conical or cylindrical structure) and not necessarily refer to ‘side’ in the same way, making comparisons between descriptions difficult. Some authors refer to dorsal and ventral sides, others to anterior and posterior, other authors divide the parapodia in four sides and most of the descriptions do not make clear which sides they are considering. On the other hand, it has been shown that parapodia are highly contractile so the display of papillae varies between a retracted parapodium with most papillae appearing distally grouped (e.g., Figs. 6H, 6J, 6K) and a relaxed parapodium with more separated papillae (e.g., Fig. 6I).

**Figure 8:**
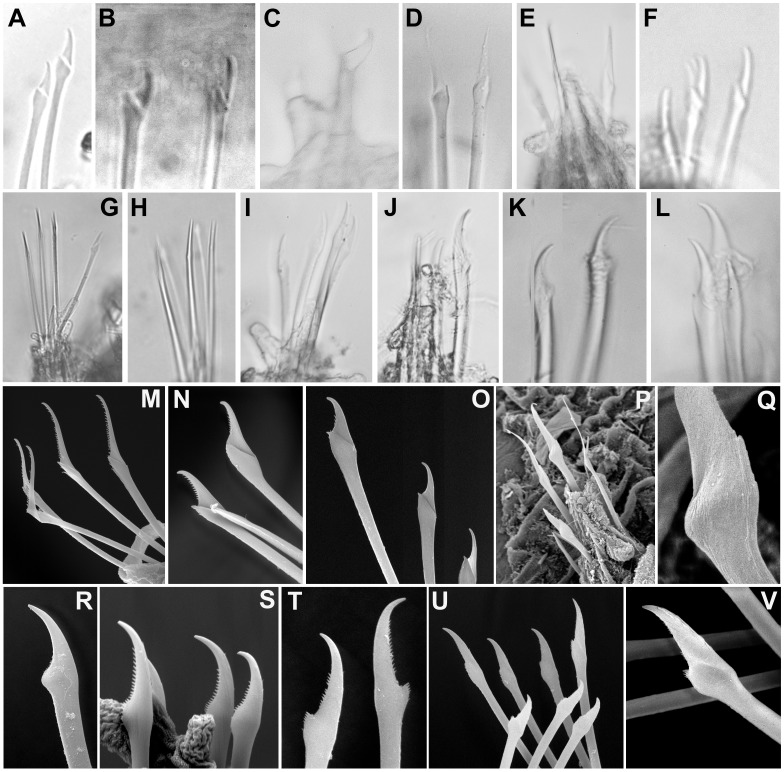
Micrographs and SEM images of chaetal details. (A) *Ephesiella antarctica,* from King George Island,** Antarctica** (SMF 24630); (B) *Ephesiella* cf. *cantonei*, from Banyuls, France (LACM AHF POLY 5372); (C) *Ephesiella mammifera*, from Baja California, Mexico (LACM AHF POLY 5413); (D) *Ephesiella muhlenhardtae*, holotype (ZHM P18941); (E) *Ephesiella pallida*, paratype (USNM 58483); (F) *Ephesiella phuketensis*, holotype (PMBC 18542); (G) *Ephesiopsis guayanae*, paratype (LACM-AHF POLY TYPE943); (H) *Sphaerodorum indutum*, holotype (LACM-AHF POLY TYPE960); (I) *Sphaerodorum papillifer*, holotype (USNM 17379); (J, K) *Sphaerodorum papillifer*, paratypes (USNM 17380); (L), *Sphaerodorum recurvatum*, paratype (LACM-AHF POLY TYPE963); (M, N, O); *Ephesiella abyssorum*, from Melilla, Mediterranean (MNCN 16.01.3457), showing variation of chaetae along segments within same specimen; (P, Q), *Ephesiella* sp. from Antarctica (MNCN 16.01.3555); (R, S, T), Chaetae variation along same specimen of *Sphaerodorum flavum*, from Snøhvit, Norway (NTNU-VM 73708); (U, V), *Sphaerodorum flavum*, from Norway (ZMBN 126047).

The range of variation in the number of parapodial papillae reported within the group varies from none to 20, but most species records indicate 4 −12. The only species considered as lacking papillae is *S. recurvatum*, but the poor condition of the only known specimen, the holotype, could be the explanation for such interpretation (Fig. 7V). The specimens with larger number of parapodial papillae are generally large. This is the case for types of *S. indutum* (Fig. 7J), *S. olgae* (Fig. 7P), *S. papillifer* (Fig. 7S) and some of the specimens of *E. abyssorum* and *S. flavum*, measuring over 10 mm, with around or over 10 parapodial papillae. Contrary, the smaller forms have lower number of papillae and this is the case for the types of *E. mixta* (Figs. 6A, 6M) and *E. australiensis*, measuring less than 2 mm and with only 2 −3 parapodial papillae. An exception to this pattern is *E. pallida*, with specimens measuring over 10 mm reported and with around four parapodial papillae (Fig. 7R). Species with high number of specimens available have shown some interspecific variability (e.g., *E. abyssorum* and *S. flavum*, Figs. 6D–6H) but these nominal taxa also show a broad bathymetrical and geographic distribution range and could well be a complex of species.

The position and arrangement of the parapodial papillae, could be one of the morphological features able to discriminate between species. Some variation between specimens from same putative species and collected in same localities has been observed and therefore it would still need to be further investigated. We encourage the use of a unanimous methodology and terminology to refer to the parapodial sides in order to minimise subjectivity (e.g., Fig. 7, as proposed by Capa & Bakken, 2015).

#### Chaetae (Figs. 8A–8V, Supplemental Information).

Not only does the presence of simple and/or compound chaetae seem not to characterise monophyletic groups (Fig. 2) but also there is no clear boundary between what should be defined as simple or compound chaetae. The shaft and blade of some *Ephesiella* species are not clearly divided (e.g., *E. brevicapitis*; Moore, 1909) and in some species of typically simple compound chaetae, a groove has been noted indicating a possible fusion of blade and shaft (e.g., *S. ophiurophoretos* and *S. olgae*; Martín & Alvà, 1988; Moreira & Parapar, 2011), and is also herein reported from specimens of *S. flavum* (Figs. 8U, 8V).

Chaetal morphology has been utilised to distinguish species. The most common traits include the relative length/width of the blades (in compound chaetae) or distal tip (in simple chaetae), the curvature of the distal end, or the presence of a tooth or knob at the distal edge of the shaft (e.g., Capa & Bakken, 2015). Intraspecific variability in most cases has not been assessed and direct examination of material has shown that a broad variation in chaetal morphology can be observed from anterior to posterior segments with a general trend that distal tips shorten and become more curved towards posterior chaetigers (e.g., Figs. 8M–8O).

*Ephesiella* species described with almost straight chaetae in all chaetigers are typically *E. bipapillata, E. macrocirris*, *E. mixta* and *E. muhlenhardtae* (e.g., Fig. 8D). The types revisited indeed showed no curved chaetae in any segment of any of these species, and blades are also proportionally longer than in other congeners, reaching up to five times the length of the oblique proximal edge, with the exception of *E. macrocirris,* where chaetae are up to four times the length of the proximal edge. Species with only strongly curved and short blades are *E. australiensis* and *E. mammifera* (Fig. 8C; Fauchald, 1974; Hartmann-Schröder, 1982; Capa & Bakken, 2015) with lengths of blades shorter than 2.5× the oblique base of the blades. Detailed study of specimens from the Mediterranean and the Norwegian Sea, potentially *E. cantonei* and *E. abyssorum* respectively, showed curved and short chaetae, but some also were longer and straighter in anterior chaetigers (Figs.  8B, 8M–8O).

Serration on one of the distal edges is often fine and difficult to assess under the compound microscope but variation has been observed within specimens under SEM (Figs. 8M–8O). Contrary to expectations, the posterior-most chaetae are often smooth, even though annelids add segments posteriorly and these chaetae should be newer and less eroded (e.g., Figs. 8M–8O).

*Ephesiopsis guayanae* has both compound and simple chaetae in every segment (Hartman & Fauchald, 1971; Fauchald, 1974) a condition verified in both holo- and paratype (Capa, Osborn & Bakken, 2016; Fig. 8G). The compound chaetae (two per parapodia in mid-body segments) has a curved blade while the simple chaetae (three per parapodia) has a straight and tapering edges tip. Simple chaetae are distally flattened and resemble those present in *S. vietnamense*, according to the original drawings as *Sphaerodorum* sp. A. (Gallardo, 1968). Some simple chaetae of *E. guayanae* seem to be the result of a fusion of the shaft and blade, with the edge between them still marked as a faint ridge (Capa, Osborn & Bakken, 2016).

Variation in the type of simple chaetae among members of *Sphaerodorum* has also been noted. Some species, such as *S. papillifer*, *S. recurvatum* and *E. vietnamense* were described to have a “spur in the subdistal swelling boss” (Fauchald, 1974) but after looking at this ‘spur’ under the SEM it turned out to be the distal serration of the shaft, and this feature is also present (but not always) in specimens identified as *S. flavum* (Fig. 8T). *Ephesiella recurvatum* was described to have strong hooks in first segment while in *S. olgae* these can be almost straight (Fauchald, 1974; Moreira & Parapar, 2011).

The number of chaetae in long-bodied sphaerodorids is fairly constant and most specimens present 3–6 chaetae per parapodium (Figs. 6C–6K, 7E–7J; Moore, 1909).

#### Presence of embryos

The reproductive mode of long-bodied sphaerodorids is unknown. References of *Ephesiella mixta* being hermaphroditic was made after finding an individual (the holotype), with eggs in anterior, sperm in posterior segments and embryos in middle segments (Fauchald, 1974). Revision of this specimen did not provide evidence about this because sperm is no longer distinguishable. The structures that seem to have been considered embryos could be the segmental ganglia (called perikarya according to Filippova et al., 2010), that are well developed in sphaerodorids (Reimers, 1933; Kuper & Purschke, 2001).

**Table:** 

***Sphaerodorum*Ørsted, 1843**
*Sphaerodorum*Ørsted, 1843: 42.
*Ephesia*Rathke, 1843: 174 −176.
*Ephesiella*Chamberlin, 1919**new synonym**.
*Ephesiopsis*Hartman & Fauchald, 1971**new synonym**.

Type species: *Sphaerodorum flavum* Ørsted, 1843.

Emended diagnosis: Body long and slender with blunt anterior end. Two longitudinal rows of macrotubercles over dorsum, one pair per segment, above parapodia. Macrotubercles sessile, with terminal papillae. Two longitudinal rows of microtubercles, with a collar and a terminal papilla, one pair per segment, running parallel between macrotubercles. Additionally, papillae arranged in 4–5 transverse rows on dorsum and ventrum. Head appendages, palps, lateral and median antennae short, spherical or digitiform. Parapodia with simple, compound or pseudocompound chaetae. Simple hooks absent or present on first chaetiger (sometimes also on second or third chaetiger).

Remarks: the emended diagnosis includes the variation of chaetal morphology (including simple, compound or pseudocompound chaetae) previously regarded as distinctive between long-body sphaerodorid genera.

The current nominal species circumscribed in *Sphaerodorum*, and nomenclatural changes proposed, after this study are the following:

*Sphaerodorum abyssorum* Hansen, 1879 **n. comb**.

Type locality: Norway, 63°5′N 3°0′E, 960 m deep.

*Sphaerodorum antarctica* (McIntosh, 1885) **n. comb**.

Type locality: Antarctica, 62°26′S, 95°44′E, 3,612 m deep.

*Sphaerodorum australiensis* (Hartmann-Schröder, 1982) **n. comb**.

Type locality: Cervantes, Western Australia, intertidal.

*Sphaerodorum bipapillatum* (Kudenov, 1987) **n. comb**.

Type locality: Louisiana, Gulf of Mexico, 28° 54′48″N, 89°59′05″W, 33.6 m deep.

*Sphaerodorum brevicapitis* Moore, 1909 **n. comb**.

Type locality: California, Eastern Pacific, 3,740 m deep.

*Sphaerodorum cantonei* (Mòllica, 1994) **n. comb**.

Type locality: Sicily, Central Mediterranean, 3–6 m deep.

*Sphaerodorum flavum* Ørsted, 1843

Type locality: Denmark, shallow water, perhaps intertidal.

*Sphaerodorum gallardi* (Fauchald, 1974) **n. comb**.

Type locality: South Vietnam, 19 m deep.

*Sphaerodorum guayanae* (Hartman & Fauchald, 1971) **n. comb**.

Type locality: Surinam, Western Atlantic, 520–550 m deep.

*Sphaerodorum indutum* Fauchald, 1974

Type locality: Antarctica, 61°25′S, 56°30′W, 300 m deep.

*Sphaerodorum macrocirris* (Hartman & Fauchald, 1971) **n. comb**.

Type locality: New England, Western Atlantic, 1,470–1,330 m deep.

*Sphaerodorum mammiferum* Fauchald, 1974 **n. comb**.

Type locality: Baja California, Eastern Pacific, intertidal.

*Sphaerodorum mixtum* (Hartman & Fauchald, 1971) **n. comb**.

Type locality: Bermuda, 3,753 m deep.

*Sphaerodorum muhlenhartdtae* (Hartmann-Schröder & Rosenfeldt, 1988) **n. comb**.

Type locality: joinville Island, Antarctica, 63°30′S, 54°15′W, 220 m deep.

*Sphaerodorum oculatum* (Imajima, 2003) **n. comb**.

Type locality: Johashima, Japan, 100 m deep.

*Sphaerodorum olgae* Moreira & Parapar, 2011

Type locality: Bellingshausen Sea, Antarctica, 400–1,799 m deep.

*Sphaerodorum ophiurophoretos* Martín & Alvà, 1988

Type locality: Pas de Calais, English Channel, intertidal.

*Sphaerodorum pallidum* (Fauchald, 1974) **n. comb**.

Type locality: South Shetland Islands, Antarctica, 1,437 m deep.

*Sphaerodorum papillifer* Moore, 1909

Type locality: California, Eastern Pacific, 914 m deep.

*Sphaerodorum phuketensis* (Bakken, 2002) **n. comb**.

Type locality: Phuket Island, Andamen Sea, 63 m deep.

*Sphaerodorum ramosae* (Desbruyères, 1980) **n. comb**.

Type locality: Plateau de Meriadzek, North East Atlantic, 47°29.2′N 8°30.7′W, 2,156 m deep.

*Sphaerodorum recurvatum* (Fauchald, 1974)

Type locality: South Africa, Indian Ocean, 29° 45′S, 31°40–39′E, 445 m deep.

*Sphaerodorum shivae* (Rizzo, 2009) **n. comb**.

Type locality: Off São Paulo, Western Atlantic, 24°07.637′S 45°51.895′ W, 147 m deep.

*Sphaerodorum vietnamense* (Fauchald, 1974)

Type locality: South Vietnam, 32 m deep.

## Discussion

### Absence of reciprocal monophyly in currently accepted genera

Phylogenetic analyses of molecular data revealed that both *Ephesiella* and *Sphaerodorum* are paraphyletic, implying also that chaetal morphology (i.e., either simple or compound chaetae) is not a distinct attribute separating genera (contrary to Pettibone, 1963 and subsequent authors). Revision of the literature and direct examination of specimens from different geographical areas and depths also exposed that classification of chaetae in discrete groups is not always possible since there is a continuum in the level of fusion of blades and shaft and intermediate forms. Consequently, and following the principle of priority in the International Code of Zoological Nomenclature (1999), *Sphaerodorum* is the first formal scientific name given and shall be considered as the valid name, and *Ephesiella* should be considered as a junior synonym. All 17 species previously considered within *Ephesiella*, should thus be transferred to *Sphaerodorum*.

Unfortunately, no fresh or ethanol fixed specimens of the monotypic *Ephesiopsis,* were available for this study, and therefore confirmation of its phylogenetic position was not possible with DNA sequence data. We advocate here for uniformity and consistency in the definition of taxonomic groups based in the morphological features and propose that all long-bodied sphaerodorids, with longitudinal rows of macrotubercles with terminal papillae and longitudinal rows of microtubercles should be considered members of the same genus. We thus also suggest the synonimization of *Ephesiopsis* with *Sphaerodorum*.

### Species diagnostic characters

Regardless of the nature of the morphological features (qualitative or quantitative, discrete or continuous, fixed or being polymorphic within species) used for discriminating between species, character states need to be distributed in different frequencies across species (Padial & De la Riva, 2010). Some of the morphological features traditionally used in long-bodied sphaerodorid species descriptions have shown uncertain validity for species discrimination because they do not follow this premise.

The absence of specific prostomial appendages, as stated in the descriptions of *Ephesiella pallida*, *E. cantonei*, *E. ramosae* and *Sphaerodorum vietnamense* (Fauchald, 1974; Desbruyères, 1980; Mòllica, 1994), are questioned. This condition was not obvious in the types examined but none of the additional material studied lacked anterior appendages. This condition (absence of median or lateral antenna) was one of the main diagnostic features for the species, and therefore its validity should be checked.

The relative length of prostomial appendages, the shape and length of parapodia, parapodial lobes and ventral cirri, the shape and size of the macro-, microtubercles and papillae and their relative arrangement above the parapodia (Hartman & Fauchald, 1971; Fauchald, 1974; Desbruyères, 1980; Moreira & Parapar, 2011) are characters that show great intraspecific (sometimes even intra-individual, along the different segments) variation, likely due to musculature contraction and character states greatly overlap between putative species. Some authors have indicated the sensory nature of the epithelial papillae, being longer toward parapodia and in posterior regions of the body, and retractile (Moore, 1909; Helm & Capa, 2015).

The discernibility of eyes and hooks is also related to the transparency of the tissue (preservation time and conditions, perhaps also age) and not being ejected or conspicuous in fixed material is not necessarily synonymous of absence (Bakken, 2002). On the other hand, hooks were verified to be lacking in specimens among populations were they are present, indicating also intraspecific plasticity (maybe individuals are able to replace them after loss) and probably not an attribute for characterising species (e.g., *Ephesiella gallardoi*), or at least with the current species definitions.

The chaetal morphology and number and arrangement of parapodial papillae do not depend on the muscle contraction, and could be, in this line, reliable features for species discrimination (Kudenov, 1987; Moreira & Parapar, 2011). Nevertheless, chaetal morphology (e.g., shape and length of blades) and the number and position of parapodial papillae show intraspecific variability that overlap between putative species. A profound revision of these features should be carried out, considering a substantial number of specimens of verified members of same species, in order to test their utility for taxonomic purposes. We also encourage the use of a unanimous methodology in respect of the position of parapodial papillae and agreed terminology to refer to the parapodial sides or faces in order to minimise subjectivity (e.g., that proposed by Capa & Bakken, 2015: Fig. 5, or shown in Fig. 7 herein). The parapodial lobes are in most cases difficult to distinguish from papillae and also hard to locate with respect to the randomly arranged chaetae. Their presence and position with respect of other parapodial structures is subjective (e.g., Fauchald, 1974), therefore is an attribute that does not seem to be useful for separating species.

For the reasons explained above, there are a few combinations of morphological characters that seem useful to discriminate between species (i.e., number and arrangement of parapodial papillae, and chaetal morphology and variation along the body segments). Others, that have been observed in only some putative species and could represent species synapomorphies (i.e., the presence of microtubercles and accompanying simple, serrated chaetae in the first chaetiger) are often not found in the species descriptions. It seems like the boundaries between species are not clearly understood and a worldwide revision of members of the long-bodied sphaerodorids is required.

### Phylogeography

The genus *Sphaerodorum* (including *Ephesiella* and *Ephesiopsis*) is considered as cosmopolitan and it has been reported from cold waters of the Arctic and Antarctic slope, and bathyal, abyssal, continental shelf and slope communities from the Atlantic and Indo-Pacific and also from tropical shallow waters. The absence of records from other worldwide localities may reflect a lack of sampling rather than an absenteeism of this group of sphaerodorids. There is little information available about the reproductive strategy or dispersal capabilities in long body sphaerodorids (Capa, Bakken & Purschke, 2014; Capa, Osborn & Bakken, 2016). Members of *Sphaerodorum* are dioicous and the sperm of *Sphaerodorum flavum* is of the ‘primitive type’ probably indicating external fertilization Franzen, 1956;  Franzen, 1958; Capa, Bakken & Purschke, 2014). Other members of the family, the short-bodied forms, have shown pseudocopulation and internal fertilization (Capa & Rouse, 2015). Dispersal capability is therefore assumed limited at these in short bodied sphaerodorids (Capa, Aguado & Bakken, 2016). However, no “sexual organs”, or sperm storage or larvae have been found in the long-bodied forms, and the question about the fertilization mode, development and dispersal capacity remains unanswered. In previous studies, fertilization has been assumed to be external, and the size of oocytes, together with the great amount of lipid droplets and yolk granules stored in them suggest direct, non-pelagic and lecithotrophic development (Christie, 1984).

## Conclusions

• Nuclear and mitochondrial DNA sequences of member of specimens identified as *Ephesiella* and *Sphaerodorum* due to the chaetal morphology) were not recovered reciprocally monophyletic, suggesting these two genera should be synonymised. We propose species of *Ephesiella*, should be transferred to *Sphaerodorum*.
• These findings provide evidence that compound or simple chaetae are not valid to split long-bodied sphaerodorids into natural groups.
• Based on only morphological data, we also suggest *Ephesiopsis* to be considered as a junior synonym of *Sphaerodorum*.
• Some of the species traditional diagnostic characters have shown not to be unique (as they show a broader intraspecific variability than previously reported or not enough differences to discriminate between species). We highlight the need of a thorough revision that assesses validity of the species.

##  Supplemental Information

10.7717/peerj.5783/supp-1 Supplemental Information 1 Supplementary material

10.7717/peerj.5783/supp-2 Supplemental Information 2 Trees Sup Mat Fig. 1.

10.7717/peerj.5783/supp-3 Supplemental Information 3 18S sequences

10.7717/peerj.5783/supp-4 Supplemental Information 4 28S sequences

10.7717/peerj.5783/supp-5 Supplemental Information 5 16S sequences

10.7717/peerj.5783/supp-6 Supplemental Information 6 COI sequences

## Additional Information and Declarations

### DNA Deposition

The following information was supplied regarding the deposition of DNA sequences: DNA fragments sequenced for this study are accessible via GenBank accession numbers MH768888 to MH768963—this information is included in Table 1.

### Data Availability

The following information was supplied regarding data availability: The raw data are supplied as a Supplemental File.
