# Supplementary material for: Three, two, one! Revision of the long-bodied sphaerodorids (Sphaerodoridae, Annelida) and synonymization of Ephesiella, Ephesiopsis and Sphaerodorum
Source: PeerJ. 2018 Oct 26;6:e5783. doi: 10.7717/peerj.5783 (PMC6204827; doi:10.7717/peerj.5783)
Supplement: Supplemental Information 1 [file peerj-06-5783-s001.docx]

**SUPPLEMENTARY MATERIAL**

Summary of supplementary material:

- Figure 1. Phylogenetic hypothesis after Bayesian inference (Mr. Mayes) and Maximum Likelihood (RAxML) analyses of different datasets. A. BI of complete dataset, after removal of alignment-ambiguous sites with gblocks; B. ML of complete dataset, after removal of alignment-ambiguous sites with gblocks; C. BI of nuclear partition and all sites; D. ML of nuclear partition and all sites; E. BI of mitochondrial partition and all sites; F. ML of mitochondrial partition and all sites.
- Material examined.
- Table 1. Comparison of features relative to prostomial appendages from types and non-type material.
- Table 2. Comparison of features relative to presence and number of prostomial eyes in type specimens and non-type material.
- Table 3. Comparison of features relative to presence and number of hooks in anterior chaetigers in type specimens and non-type material.
- Table 4. Comparison of features relative arrangement of macro- and microtubercles from segment 2 in type and non-type specimens.
- Table 5. Comparison of features relative to the number and shape of parapodial papillae in type and non-type specimens.
- Table 6. Comparison of features relative to the number and shape of chaetae in type and non-type specimens.

**Material examined**:

***Sphaerodorum abyssorum* Hansen, 1878**. *Holotype*: ZMBN 1972, Norwegian North-Atlantic Expedition 1876-78, stn 33, 63º5'N 3º0'E, 960 m, 30 June 1876. *Additional material*: SMF24631, Irminger Basin, South Iceland, 63°00'27"N 28°04'05"W, 1594 m (1 ind.); SMF 24632, Irminger Basin, South Iceland, 63°00'27"N 28°04'05"W, 1594 m (1 ind.); ZMBN 115498, Skagerrak, 58°02'11"N 9°30'15"E, 406 m (1 ind.); ZMBN 115503, Varangerfjord, Barents Sea, Norway, 70°49'54"N 28°31'03E, 304 m; ZMBN 125428, Norwegian Sea, 67°48'16"N 9°41'13"E, 823 m, (1 ind.); SMF 24633 (1 ind.), SMF 24634 (1 ind.) and SMF 24635 (1 ind.), Denmark Strait, East Greenland, 67°50'47"N 23°41'45"W, 1250 m; NTNU-VM 73254, Trøndelag, Agdenes, 63°37'37"N 9°37'54"E, 45 m (1 ind.); ZMBN 115523, Oslofjord, Drøbak, Norway, 59°39'01"N 10°37'09"E, 30 m (1 ind.); ZMBN 125840, Kvamsøya, Norway, 62°12'18"N 5°26'42"E, 85 m (1. ind.); ZMBN 125842, Barents Sea, Norway, 71°16'31"N 27°00'56"E, 276 m, (1 ind.); ZMBN 126045, off Nordland, Norway, 67°57'20"N 9°35'33"E, 1299 m (i ind.); MNHN A421, Mediterranean Sea, France, Tatihou, Isla in Saint-Vaast-la-Hougue. Coll: 1901 (1 ind.).

***Sphaerodorum antarctica* (McIntosh, 1885)**. MNCN 16.01/3554, S. Livingston, South Shetland Islands, 62°45,1610’S 60°33, 2028’W, 429 m (2 ind.); MNCN 16.01/3555, S. Livingston, South Shetland Islands, 62°41,8578’S 60°31,6035’W, 263 m (2 ind.); USNM 46565, Antarctic Ocean, Weddell Sea 74°28'05"S 30°31'41"W, 513 m (1 ind); SMF 24630 (SPH 032), from King George Island, Antactica.

***Sphaerodorum australiensis* (Hartmann-Schröder, 1982)**. *Holotype*: ZMH P-16773 Cervantes, Western Australia, in fine sand and *Posidonia*; Paratype: ZMH P-17448, same locality as holotype (1 ind.). *Additional material*: Western Australia: AM W.42699 (1 ind.), North West Shelf, 19° 28' S, 118° 55' E, 39 m (1 ind.); AM W.42700 (1 spec.), 2 km west of Angel Island, Dampier Archipelago, 20° 29' 46" S, 116° 47' 29" E, 10 m, (1 ind.); AM W.42701, Angel Island, Dampier Archipelago, 20° 27' 41" S, 116° 47' 31" E, 14 m, (2 ind.); AM W.42702 (2 ind.), south west Enderby Island, 20° 37' 18" S, 116° 27' 23" E, 14 m, (2 ind.); AM W.42703 (2 ind.), north west end Legendre Island, 20° 21' 13" S, 116° 50' 26" E, 21 m (2 ind.); AM W.42704 (4 ind.), 1 km north east of Delambre Island, Dampier Archipelago, 20° 25' 43" S, 117° 05' 07" E, 14 m, (4 ind.); NMV F.162479 (1 ind.), off Pelsart Island, near Geraldton, 29° 00' 10" S, 113° 46' 26" E, 409 m (1 ind.). Northern Territory: NTM W.20663, Arafura Sea, 9°18'13.5"S 133°41'28.8"E, 187 m (1 ind.).

***Sphaerodorum bipapillatum* (Kudenov, 1987)**. *Holotype*: USNM 102789, Atlantic Ocean, Gulf of Mexico, USA, Louisiana, 28°56’06”N, 90°0’30”W, 33.6 m. *Paratype*: USNM 102790, Atlantic Ocean, Gulf of Mexico, USA Louisiana, 28°54’48”N, 89°59’05”W, 33.6 m (1 ind.).

***Sphaerodorum brevicapitis* Moore, 1909**. *Holotype*: USNM 17378, Pacific Ocean, USA, California, Off Santa Catalina Islands, 3740 m. *Additional material*: LACM-AHF POLY 5389, California, San Diego County, Coronado Canyon, North Coronado Island, 32°30’42”N, 117°21’37”W, 794 m, green mud (1 ind.); LACM-AHF POLY 5390, California, Los Angeles County, Santa Catalina Island, 33°23’10”N, 118°29’38”W, 86 m, gray clayey mud (1 ind.); LACM-AHF POLY 5391, California, Santa Barbara County, 12.7 miles from Point Conception Light, 34°27’25”N, 12°12’55”W, 17.4 m (2 specs); LACM-AHF POLY 5392, Baja California, near Isla Natividad Light, 27°24'00"N, 115°12'15”W aprox. 2478 m, green mud (5 specs); LACM-AHF POLY 5411, California, off Santa Cruz Island, 4°02'49”N 119°39'18”W 85 m (1 ind.); LACM-AHF POLY 5412, California, Southern California Bight, 34°00'50”N 118°35'31”W, 34 m (1 ind.).

***Sphaerodorum cantonei* (Mòllica, 1994)**. Holotype not accessible. Additional material: LACM AHF 5370, Mediterranean Sea, France, Banyuls-Sur-Mer, off Cap Oullestrel, 42°49’N, 03°08’E, 20 m (1 ind.); LACM AHF 5371, off Laboratoire Arago, 42°28’51.52”N, 03°08’14.07”E, 0.3-0.6 m (1 ind.); LACM AHF 5372, off Cap Oullestrel, 42°49’N, 03°08’E, 20 m (1 ind.); LACM AHF 5373, off Laboratoire Arago, 42°28’52.38”N, 03°08’13.84”E, 1 m (1 ind.); MNCN 16.01/3448, Mediterranean sea, Spain, Málaga (2 ind.); MNCN 16.01/3450, Mediterranean sea, Spain, Melilla (5 ind.); MNCN 16.01/3457, Mediterranean sea, Spain, Málaga, 8 m (19 ind.).

***Sphaerodorum flavum* Ørsted, 1843.** *Type material* probably lost. *Additional material*: ZMBN 125429, Norwegian Sea, 67°43'29"N 10°16'28"E, 219 m (1 ind.); ZMBN 115515, Norwegian Sea, Norway, Skjoldryggen, 65°30'02"N 6°16'13"E, 319 m (1 ind.); ZMBN 125431, Barents Sea, Norway, Varanger, 70°17'47"N 31°18'49"E, 217 m (1 ind.); ZMBN 115538, Svalbard, Norway, Storfjord, 78°38'50.8"N 20°57'42.5"E, 74 m (1 ind); ZMBN 125430, Norwegian Sea, Norway, Sognefjorden, 103 m (1 ind.); ZMBN 125840, Norwegian Sea, Norway, Kvamsøya, 62°12'18"N 5°26'42"E, 85 m (1 ind.); ZMBN 126043, Labrador Sea, Greenland, 60°00'00"N 46°31'12"W, 167 m (1 ind.); ZMBN 126044, Greenland Sea, Greenland, 63°33'00"N 39°18'00"W, 213 m (1 ind.); ZMBN 126046, Norwegian Sea, Norway, Nordland, 67°43'19"N 10°16'30"E, 219 m, (1 ind.); ZMBN 126045, Norwegian Sea, Norway, off Nordland, 67°57'20"N 9°35'33"E, 1299 m (1 ind.); MNCN 16.01/13265, Atlantic Ocean, Spain, A Coruña, 43º48'25''N 08º51'27''W (4 ind.).

***Sphaerodorum gallardoi* (Fauchald, 1974)**. *Holotype*: AHF POLY 934, Bay of Nha Trang, South Vietnam, stn 259, 1 mile SE of Grand Banc, 19 m, sandy mud.

***Sphaerodorum guayanae* (Hartman & Fauchald, 1971).** *Holotype*: LACM-AHF Poly 942, North Atlantic, off Dutch Guiana (Surinam), 7°52'00"N 54°31'30"W to 7°55'00"N 54°35'00"W, 520-550 m, 25 Apr 1963. *Paratype*: LACM-AHF Poly 943, same locality as holotype. *Additional material*: USNM 1001772, Georges Bank, 40°57'12"N 66°13'40"W (2 ind.); USNM 1001773 off New Jersey, 38°35'58"N 72°52'51"W, 2195 m (1 ind.); USNM 1001777, off New Jersey, United States, 2150 m (1 ind.); USNM 1001713, off Cape Lookout, North Carolina, 34°11'09"N 75°38'58"W, 2006 m, (1 ind.); USNM 1001780, off Delaware, 37°51'34"N 73°19'54"W, 2100 m (1 ind.); USNM 1001781, off New Jersey, 38°29'16"N 72°42'06"W, 2507 m (1 ind.); USNM 1001782, off New Jersey, 38°29'13"N 72°42'11"W, 2505 m (1 ind.); USNM 1001783, off New Jersey, 38°29'13"N 72°42'11"W, 2505 m (1 ind.); USNM 1001789, Baltimore Canyon, Maryland, 37°53'45"N 73°44'45"W, 1499 m (1 ind.).

***Sphaerodorum indutum* Fauchald, 1974**. *Holotype:* USNM 58481 Antarctic Ocean, South Shetland Islands, west Aspland Island, 61°25'01”S 56°31'01”W, 300 m. *Additional material*: LACM-AHF Poly 5403 South Orkney Islands, 60°35’S, 40°44’W to 60°34’S, 40°44’W, 631-641 m (12 ind.); LACM-AHF Poly 5404, Deception Island, Bransfield Strait, South Shetland Islands, 62º50’S, 60º40’W to 62º51’S, 60º35’W, 267-311 m (6 ind.); LACM-AHF Poly 5405, Bransfield Strait, 61°18'S, 56°09'W to 61°20'S, 56°10'W, 220-240 m (6 ind.); LACM-AHF Poly 5406, off Peter I Island, Bellingshausen Sea 70°20’S, 99°10’W to 70°13’S, 98°57’W, 3848-3980 m (1 ind.); LACM-AHF Poly 5407, South Orkney Islands, 60°50’S, 42°55’W to 60°52’S, 42°56’W, 298-302 m (12 ind.).

***Sphaerodorum macrocirris* (Hartman & Fauchald, 1971)**. *Holotype*: LACM-AHF POLY 936, New England continental slope, USA, 39°46'30”N 70°43'18”W, 1470-1330 m. *Paratypes*: LACM-AHF POLY 937 (2 ind.), same locality as holotype.

***Sphaerodorum mammifera* Fauchald, 1974**. *Holotype*: LACM-AHF POLY 935, Hancock Cove, 20 miles east of Punta Eugenia, Baja California, Mexico, 27°48'47”N, 114°43'07”W, 79 m. *Additional material*: LACM-AHF POLY 5413, at Old English Mill, Bahia de San Quintin, Baja California, 30°29’03”N, 115°58’38”W (1 ind.).

***Sphaerodorum mixta* (Hartman & Fauchald, 1971)**. *Holotype***:** LACM-AHF POLY 940, northwest of Bermuda, 38°33'N, 68°32'W, 3753 m, 17 Dec 1965. No paratypes or additional material found to date.

***Sphaerodorum muhlenhardtae* (Hartmann-Schröder & Rosenfeldt, 1988)**. *Holotype*: ZMH P.18941 (wrongly referred to P.18949 in the original description); *Paratypes*: ZMH P.19150, Joinville, 62°05'18”S 57°39'00”W, 265 m (2 ind.); ZMH P.19151, Bransfield Strait, 63°11'18”S 58°47'00”W, 93 m (1 ind.); ZMH P.19152, Point Thomas, 200 m (1 ind.). *Additional material*: ZMH P.25979 Antarctica, XV/3 PS 48 ST. 188 (2 ind.).

***Sphaerodorum oculata* (Imajima, 2003).** No material examined, except of a specimens presumably belonging to this species as *Ephesiella* cf. *oculata*, from Japan, UUZM 54541, from Sagami Bay, Japan, 13 Jun 1914.

***Sphaerodorum olgae* Moreira & Parapar, 2011**. *Holotype*: MNCN 16.01/13158, Belingshausen Sea, 431-1799 m. *Paratypes*: MNCN 16.01/13159 (1 ind.), MNCN 16.01/13160 (1 ind.), MNCN 16.01/13161 (3 ind.), MNCN 16.01/13162 (1 ind.), all paratypes from same sample as holotype.

***Sphaerodorum ophiurophoretos* Martín & Alvà, 1988.** No material examined.

***Sphaerodorum pallidum* (Fauchald, 1974)** *Holotype*: USMN 58482, Antarctic Ocean, South Shetland Islands, 62°02'31”S 61°08'31”W, 1437 m. *Paratypes*: USNM 58483, same sample. *Additional material*. USNM 56616, Antarctic Ocean, 62°42'00”S 56°12'00”W, 494–507 m (2. ind.); USNM 56617, Antarctic Ocean, 61°48'00”S 61°12'00”W, 4758 m (3 ind.); USNM 56618, Antarctic Ocean, 61°48'00”S 61°12'00”W, 1180 m (1 ind.); USNM 56619, Antarctic Ocean, 64°54'00”S 68°18'00”W 412 m, (1 ind); USNM 56620, Antarctic Ocean, 2°42'00”S 54°42'00”W, 210–220 m (1 ind.).

***Sphaerodorum papillifer* Moore, 1909.** *Holotype*: USNM 17379, off San Clemente Island, San Diego, California, USA, 914 m, green mud. *Paratypes*: USNM 17380, same locality as holotype (3 ind.).

***Sphaerodorum phuketensis* (Bakken, 2002).** *Holotype* PMBC 18542, Andaman Sea, Thailand, 7°30'N 98°22'E, 63 m. *Paratype* PMBC 18541, Andaman Sea, Thailand, 6°45'N 99°21'E, 38 m (1 ind.).

***Sphaerodorum ramosae* (Desbruyères, 1980).** *Holotype*: MNHN POLY TYPE 6663, Plateau de Meriadzek, 47°29'12”N 8°30'42”W, 2156 m.

***Sphaerodorum recurvatum* Fauchald 1974.** *Holotype*: LACM-AHF POLY TYPE 962, off Durban, Natal, South Africa, Indian Ocean, 29°45' S, 31°40’E to 29°45’S, 31°39' E, 445-430 m, *Paratypes*: LACM-AHF POLY TYPE 962, same locality as holotype (2 ind.).

***Sphaerodorum shivae*** **(Rizzo, 2009).** *Holotype*: MZSP883, off Santos, São Paulo State, Brazil, 24°07'38”S 45°51'53”W, 147 m, 09 Jan 1998. *Paratypes*: MZSP1031, off Santos, São Paulo State, Brazil, 24°07'38”S 45°51'53”W, 147 m, 09 Jan 1998 (2 ind.).

***Sphaerodorum vietnamense* Fauchald 1974.** *Holotype*: LACM-AHF POLY TYPE 961, Bay of Nha Trang, 2 miles south of Mui Tre, Hon Lo, North Pacific, South China Sea, South Vietnam, 12°14’39”N, 109°19’43”E, 32 m, slightly muddy sand with pebbles.

Table 1. Comparison of features relative to prostomial appendages from types and non-type material. Abbreviations and symbols: ma, median antenna; -, not applicable, not mentioned or not observed.

|  | **NUMBER** | | | |  | **RELATIVE LENGTH** | | | |
| --- | --- | --- | --- | --- | --- | --- | --- | --- | --- |
|  | **Original description** | **Revision type material** | **Other records** | **Revision additional material** |  | **Original description** | **Revision type material** | **Other records** | **Revision additional material** |
| *Sphaerodorum abyssorum* | 5 | 5 | 5^1^ | 5 |  | ma shorter | ma shorter | am shorter^1^ | am shorter- similar |
| *S. antarctica* | 4? | - | 5^2^ | 5 |  | ? | - | am longer^2^, am shorter^3^ | am shorter |
| *S. australiensis* | 5 | 5 | - | 5 |  | ma shorter | ma shorter | - | am shorter |
| *S. bipapillata* | 5 | 4? | - | - |  | ma shorter | not seen | - | - |
| *S. brevicapitis* | 5 | 5 | 5 | 5 |  | all small | all small | am shorter^4^ | am shorter |
| *S. cantonei* | 4 | - | - | 5 |  | ma absent | - | - | am shorter |
| *S. gallardoi* | 5 | 5 | - | - |  | ma shorter | ma shorter | - | - |
| *S. macrocirris* | 5 | 5 | - | 5 |  | ma shorter | similar | - | am shorter- similar |
| *S. mammifera* | 5 | 5 | - | 5 |  | ma shorter | similar | - | am shorter |
| *S. mixta* | 5 | 5 | - | -- |  | ma shorter | ma shorter | - | - |
| *S. muhlenhardtae* | 5 | 5 | 5 | 5 |  | similar | similar | similar | am shorter- similar |
| *S. oculata* | 5 | - | - | 5 |  | ma shorter | - | - | not distinguished |
| *S. pallida* | 3 | not seen | - | 5 |  | ma thicker | not seen | - | am shorter |
| *S. phuketensis* | 5 | 5 | - | - |  | ma shorter | ma shorter | - | - |
| *S. ramosae* | 4 | 2 ? | - | - |  | ma not seen | not seen | - | - |
| *S. shivae* | 5 | - | - | - |  | ma shorter | - | - | - |
| *S. guayanae* | 5 | 5 | - | - |  | ma shorter | ma slightly shorter | - | - |
| *S. flavum* | 5 | 5 | 5 | 5 |  | all small | all small | similar^1^ | am shorter- similar |
| *S. indutum* | 5 | 5 | - | 5 |  | ma longer | ma shorter | - | am shorter |
| *S. olgae* | 5 | 5 | - | 5-7 |  | ma shorter | ma shorter | - | am shorter |
| *S. ophiurophoretos* | 5 | - | - | - |  | ma shorter | - | - | - |
| *S. papillifer* | 5? | 5 | - | 5 |  | similar | not seen | - | am shorter |
| *S. recurvatum* | Prostomium missing | | - | - |  | Prostomium missing | | - | - |
| *S. vietnamense* | 4 | not seen | - | - |  | ma absent | not seen | - | - |

Literature consulted: ^1^ Moreira, 2012; ^2^ Fauchald, 1974; ^3^ Hartmann-Schröder, 1972; ^4^ Fauchald, 1972.

Table 2. Comparison of features relative to presence and number of prostomial eyes in type specimens and non-type material. Abbreviations and symbols: a/p, absent or present; -, not applicable, not mentioned or not observed; HT, hototype; PT, paratype.

|  | **Original description** | **Revision type material** | **Other records** | **Revision additional material** |
| --- | --- | --- | --- | --- |
| *Sphaerodorum abyssorum* | ? | - | 2 pairs^1^ | a/p |
| *S. antarctica* | - | - | - | - |
| *S. australiensis* | 0 | - | - | a/p |
| *S. bipapillata* | 1 pair | - | - | - |
| *S. brevicapitis* | 1 pair | - | 1 pair^2^ | - |
| *S. cantonei* | 2 pairs (fused) | - | - | 2 pairs |
| *S. gallardoi* | - | - | - | - |
| *S. macrocirris* | 2 pairs | - | - | - |
| *S. mammifera* | - | - | - | - |
| *S. mixta* | - | - | - | - |
| *S. muhlenhardtae* | 2 pairs (fused) | 2 pairs (fused) | - | a/p |
| *S. oculata* | 2 pairs | - | - | 2 pairs |
| *S. pallida* | ? in HT, 1 pair in PT | - | - | - |
| *S. phuketensis* | - | 2 pairs (fused), - in PT | - | - |
| *S. ramosae* | - | - | - | - |
| *S. shivae* | 1 pair | - | - | - |
| *S. guayanae* | absent | - | - | - |
| *S. flavum* | - | - | 2 pairs^1,3^ | a/p |
| *S. indutum* | - | - | - | - |
| *S. olgae* | - | - | - | - |
| *S. ophiurophoretos* | 2 pairs | - | - | - |
| *S. papillifer* | >2 pairs | - | - | a/p |
| *S. recurvatum* | - | - | - | - |
| *S. vietnamense* | - | - | - | - |

Literature consulted: ^1^ Moreira, 2012; ^2^Kudenov, 1994; ^3^Knigth-Jones et al., 1995.

Table 3. Comparison of features relative to presence and number of hooks in anterior chaetigers in type specimens and non-type material. Abbreviations and symbols: a/p, absent or present; -, not applicable or not mentioned; 0, not observed; HT, hototype; PT, paratype.

|  | **Original description** | **Revision type material** | **Other records** | **Revision additional material** |
| --- | --- | --- | --- | --- |
| *Sphaerodorum abyssorum* | - | 0 | not seen ^1^ | a/p |
| *S. antarctica* | - | - | 1 pair ^2^ | a/p (1 pair on each of first two segments) |
| *S. australiensis* | absent | 0 | - |  |
| *S. bipapillata* | 1 pair | 0 in HT, 2 on same parapodium in PT | - | - |
| *S. brevicapitis* | 1 pair | 0 | 1 pair ^3^ | 1 pair |
| *S. cantonei* | 1 pair | - | - | a/p |
| *S. gallardoi* | absent | absent | - | - |
| *S. macrocirris* | 2-3 pairs | 1 pair in all types | - | - |
| *S. mammifera* | 2 pairs | not seen in HT | - | 1 pair |
| *S. mixta* | 2 pairs | 1 pair | - | - |
| *S. muhlenhardtae* | 1 pair | 1 pair | - | 1 pair |
| *S. oculata* | absent | - | - | 1 pair |
| *S. pallida* | 1 pair | 1 pair in HT, 0 in PT | - | 2 pairs (some in two chaetigers) |
| *S. phuketensis* | 3 hooks | 3 hooks in PT | - | - |
| *S. ramosae* | 1 pair | 1 pair | - | - |
| *S. shivae* | 1 pair | 2 pairs in HT, 0 in PT | - | - |
| *S. guayanae* | 1 pair | - | - | - |
| *S. flavum* | 1 pair |  | 1 pair ^1^ | a/p |
| *S. indutum* | 2 pairs | 1 pair | - | not seen |
| *S. olgae* | 1 pair | 1 pair | - | 1 pair |
| *S. ophiurophoretos* | 1 pair | - | - | - |
| *S. papillifer* | - | embeded in HT, 2 pairs in one PT | - | 1-2 pairs |
| *S. recurvatum* | - | 0 | - | - |
| *S. vietnamense* | absent | 0 | - | - |

Literature consulted: ^1^ Moreira, 2012; ^2^ Fauchald, 1974; ^3^ Kudenov, 1994.

Table 4. Comparison of features relative arrangement of macro- and microtubercles from segment 2, in type and non-type specimens. Abbreviations and symbols: a/p, absent or present; -, not applicable, not mentioned or not observed; HT, holotype; mc, microtubercle, PT, paratype.

|  | **Original description** | **Revision of type material** | **Other records** | **Additional material reviewed** |
| --- | --- | --- | --- | --- |
| *Sphaerodorum abyssorum* | - | separated | separated ^1^ | separated |
| *S. antarctica* | - | separated |  | separated |
| *S. australiensis* | separated | separated | separated ^2^ | separated |
| *S. bipapillata* | separated | separated | - | - |
| *S. brevicapitis* | separated | separated | Separated ^3^ | - |
| *S. cantonei* | separated | - | - | small gap |
| *S. flavum* | separated | separated | separated ^1^ | some in contact |
| *S. gallardoi* | - | separated | - | - |
| *S. guayanae* | - | - | - | - |
| *S. indutum* | - | in contact | - | close |
| *S. macrocirris* | *-* | - | - | - |
| *S. mammifera* | partially fused | mc not seen in HT | - | some in contact |
| *S. mixta* | separated | separated | - | - |
| *S. muhlenhardtae* | separated | separated | - | separated |
| *S. oculata* | separated | - | - | some in contact |
| *S. olgae* | separated | separated | - | separated |
| *S. ophiurophoretos* | separated | - | - | - |
| *S. pallida* | separated? | mc no seen in HT or PT | - | separated |
| *S. papillifer* | - | separated | - | some in contact |
| *S. phuketensis* | in contact | in contact | - | - |
| *S. ramosae* | separated | separated | - | - |
| *S. recurvatum* | - | - | - | - |
| *S. shivae* | - | separated | - | - |
| *S. vietnamense* | - | separated | - | - |

Literature consulted: ^1^ Moreira, 2012; ^2^ Capa & Bakken, 2015; ^3^ Kudenov, 1994.

Table 5. Comparison of features relative to the number and shape of parapodial papillae in type and non-type specimens. Abbreviations and symbols: -, not applicable, not mentioned or not observed; HT, hototype; PT, paratype.

|  | **NUMBER** | | | | |  | **SHAPE AND RELATIVE LENGTH** | | | |
| --- | --- | --- | --- | --- | --- | --- | --- | --- | --- | --- |
|  | **Original description** | **Revision of type material** | **Other records** | **Additional material reviewed** | |  | **original description** | **revision type material** | **Other records** | **Additional material reviewed** |
| *Sphaerodorum abyssorum* | *-* | 4-6 | 8-9 ^1^ | 4-9 | |  | - | all similar | all similar ^1^ | all similar |
| *S. antarctica* | many | - | none? ^2^ | | - |  | - | - |  |  |
| *S. australiensis* | 2-3 | 6 | 6^3^ | - | |  | - |  |  |  |
| *S. bipapillata* | 9 | ~6 (8 in one PT) | - | - | |  | all similar | all similar | - | - |
| *S. brevicapitis* | > 20 drawn | 1 dorsal (?) | 4 ^4,5^ | | - |  | - | - | - | - |
| *S. cantonei* | >4 | - | - | 6 | |  | - | - | - | - |
| *S. flavum* |  |  | 10-12 ^7^, 12 ^1^ | 10-20 | |  | - | all similar | all similar |  |
| *S. gallardoi* | 4-5 on each face | 4 | - | - | |  | - |  |  |  |
| *S. guayanae* | - | - | - | - | |  | - |  |  |  |
| *S. indutum* | 15-20 | > 15 | - | ~15 | |  | large | all similar | large ^7^ | all similar |
| *S. macrocirris* |  | ~10 | > 5 on each face ^2^ | | - |  | - |  | - | all similar |
| *S. mammifera* | ~10 | ~5 in HT | - | 10 | |  | - |  | - | all similar |
| *S. mixta* | 2 | 3 | 1? ^2^ | - | |  | large papilla on sup. edge | all similar | - | - |
| *S. muhlenhardtae* | >4 | ~10 | 5-6 ^6^ | 9 | |  | all similar | all similar | - | - |
| *S. oculata* | 3-4 on each face | - | - | 6-7 | |  | - |  |  | acicular lobe longer |
| *S. olgae* | 9-11 | 9-11 | - | 9-11 | |  | ventral spherical | - | - | - |
| *S. ophiurophoretos* | < 10 in drawings | - | - | - | |  | all small | - | - | - |
| *S. pallida* | 2 anterior and 2 posterior | 4-5 | 4 ^7^ | | - |  | - | all similar | - | - |
| *S. papillifer* | > 12 drawn | ~8 | - | ~10 | |  | longer distally |  | small ^2^ | all similar |
| *S. phuketensis* | numerous | 6-8 | - | - | |  | - |  |  |  |
| *S. ramosae* | 6-8 | 5-7 | - | - | |  | basal longer than distal | basal longer | - | - |
| *S. recurvatum* | Absent (?) | - | - | - | |  | - | - | - | - |
| *S. shivae* | 7? (in drawing) | 3-5 | - | - | |  | - | all similar | - | - |
| *S. vietnamense* | - | 5 | - | - | |  | - | - | - | - |

Literature consulted: ^1^Moreira, 2012; ^2^Fauchald, 1974; ^3^Capa & Bakken, 2015;^, 4^ Fauchald, 1972; ^5^Kudenov, 1994; ^6^Hartmann-Schröder & Rosenfeldt, 1988; ^7^ Moreira & Parapar, 2011.

Table 6. Comparison of features relative to the number and shape of chaetae in type and non-type specimens. Abbreviations and symbols: -, not applicable, not mentioned or not observed; HT, hototype; PT, paratype.

|  | **LENGTH OF BLADES OR DISTAL TIPS / MAX WIDTH** | | | |  |  | | |  | **SHAPE OF DITAL END** | | | |  | |  | | |  |
| --- | --- | --- | --- | --- | --- | --- | --- | --- | --- | --- | --- | --- | --- | --- | --- | --- | --- | --- | --- |
|  | **Original description** | **Revision of type material** | **Other records** | **Additional material reviewed** |  | **GENERAL SHAPE** | | **Original description** | | | **Revision of type material** | **Other records** | | | | | **Additional material reviewed** | | |
| *Sphaerodorum abyssorum* | - | 4-5, gradation | 4-5, gradation | 3-5, gradation |  | compound | - | | | straight | | | - | | slightly curved | | | | |
| *S. antarctica* | - | - | 5^1, 2^ | 3-5, gradation |  | compound | - | | | - | | | straight | | straight | | | | |
| *S. australiensis* | short | 1,5-2 | - | 3-4, gradation |  | compound |  | | | strongly curved | | | variable^3^ | | variable | | | | |
| *S. bipapillata* | 3-4 (drawings) | 4-5 | - | - |  | compound | slightly curved (drawings) | | | straight | | | - | | - | | | | |
| *S. brevicapitis* | 3-4 (drawings) | all broken | long and short ^1^ | - |  | compound/ pseudo-compound | slightly curved | | | all broken | | | curved and straight ^4^ | | | | | - | |
| *S. cantonei* | 1,5-3 (drawings) | - | - | 2-5, gradation |  | compound | curved (drawings) | | | - | | | - | | curved | | | | |
| *S. flavum* | - | 4-5 | 3 ^2,5^ | 3-5 |  | simple | slightly curved (drawings) | | | curved | | | curved | | curved | | | | |
| *S. gallardoi* | 2-3 | all broken | - | - |  | compound | two types, with more or less inflated shafts and curved blades | | | all broken | | | - | | - | | | | |
| *S. guayanae* | short, 2-3 (compound) | - | - | - |  | simple | curved | | | - | | | - | | - | | | | |
| *S. indutum* | - |  | - | - |  | simple | slightly curved | | | slightly curved | | | - | | - | | | | |
| *S. macrocirris* | - | 3-4 | - | - |  | compound | not mentioned | | | straight | | | - | | - | | | | |
| *S. mammifera* | short | 2-3 | - |  |  | compound | strongly curved | | | strongly curved | | | - | |  | | | | |
| *S. mixta* | 4-5 (drawings) | 4-5 | - | - |  | compound | slightly curved | | | straight | | | - | | - | | | | |
| *S. muhlenhardtae* | 4-5 (drawings) | 4-5 | - | - |  | compound |  | | | straight | | | - | |  | | | | |
| *S. oculata* | 1-2 | - | - | - |  | compound | straight and curved (drawings) | | | - | | | - | | - | | | | |
| *S. olgae* | - | all similar | - | all similar |  | Simple/ pseudo-compound | slightly curved | | | slightly curved | | | - | | slightly curved | | | | |
| *S. ophiurophoretos* | gradation | - | - | - |  | simple/ pseudo-compound | slightly curved | | | - | | | - | | - | | | | |
| *S. pallida* | 5 (drawings) | 3 | - | - |  | compound | slightly curved | | | straight | | | - | | - | | | | |
| *S. papillifer* | gradation | gradation | - | - |  | simple | slightly curved, with a spur | | | slightly hooked | | | - | | - | | | | |
| *S. phuketensis* | 5 (drawings) | 4-5 | - | - |  | compound | slightly curved (drawings) | | | slightly curved | | | - | | - | | | | |
| *S. ramosae* | short | 3-4? | - | - |  | compound | - | | | slightly curved | | | - | | - | | | | |
| *S. recurvatum* | all similar | all similar | - | - |  | simple | strongly curved, with a spur | | | slightly hooked, with spurs | | | - | | - | | | | |
| *S. shivae* | 2-3 (drawings) | 2-3 | - | - |  | compound | curved (drawings) | | | slightly curved | | | - | | - | | | | |
| *S. vietnamense* | two types | two types | - | - |  | simple | more or less curved and with or without spur | | | slightly hooked, with spur in some | | | - | | - | | | | |

Literature consulted: ^1^ Hartman,1964; ^2^ Fauchald, 1974; ^3^Capa & Baken, 2015; ^4^Kudenov, 1994; ^5^Fauvel, 1923.
