# Supplementary material for: Three, two, one! Revision of the long-bodied sphaerodorids (Sphaerodoridae, Annelida) and synonymization of Ephesiella, Ephesiopsis and Sphaerodorum
Source: PeerJ. 2018 Oct 26;6:e5783. doi: 10.7717/peerj.5783 (PMC6204827; doi:10.7717/peerj.5783)
Supplement: Supplemental Information 2 [file peerj-06-5783-s002.pdf]

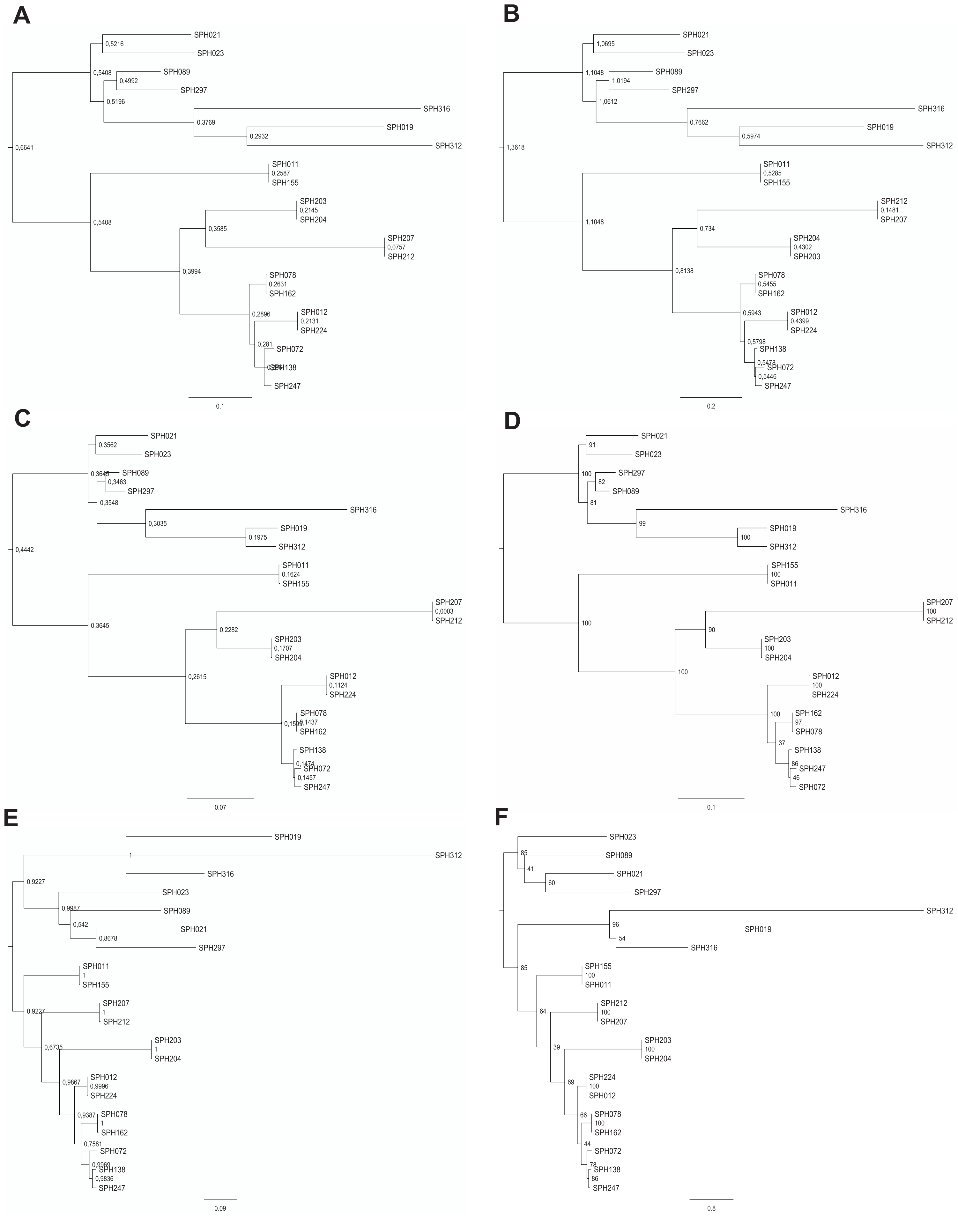

SPH078: *Ephesiella* sp., Iceland Basin, South Iceland, 2749 m
SPH162: *Ephesiella* sp., Iceland Basin, South Iceland, 2749 m
SPH203: *Ephesiella* sp., Banyuls, France, 25 m
SPH204: *Ephesiella* sp., Banyuls, France, 25 m
SPH045: *Ephesiella* sp., Irminger Basin, South Iceland, 1594 m
SPH080: *Ephesiella* sp., Irminger Basin, South Iceland, 1594 m
SPH207: *Ephesiella* sp., Skagerrak, 406 m
SPH212: *Ephesiella* sp., Finnmark, Barents Sea, 304 m
SPH011: *Ephesiella* sp., Nordland, Norway, 823 m
SPH155: *Ephesiella* sp., Denmark Strait, East Greenland, 1248 m
SPH164: *Ephesiella* sp., Trondelag, Norway, ~ 80 m
SPH232: *Ephesiella* sp., Skagerrak, Norway, 30 m

SPH072: *Sphaerodorum* sp., Sogn, Norway, 103 m
SPH247: *Sphaerodorum* sp., Svalbard, 74 m
SPH138: *Sphaerodorum* sp., Barents Sea, Norway, 217 m
SPH012: *Sphaerodorum* sp., Nordland, Norway, 219 m
SPH224: *Sphaerodorum* sp., Trondelag, Norway, 319 m
SPH019: *Clavodorum atlanticum*
SPH312: *Sphaerodoridium fauchaldi*
SPH316: *Sphaerodoridium minutum*
SPH089: *Sphaerodoropsis philippi*
SPH297: *Sphaerodorephesia* sp.
SPH021: *Sphaerodoropsis martinae*
SPH023: *Sphaerodoropsis* cf. *longianalpapilla*
